# Neuronal Ndst1 depletion accelerates prion protein clearance and slows neurodegeneration in prion infection

**DOI:** 10.1371/journal.ppat.1011487

**Published:** 2023-09-25

**Authors:** Patricia Aguilar-Calvo, Adela Malik, Daniel R. Sandoval, Christopher Barback, Christina D. Orrù, Heidi G. Standke, Olivia R. Thomas, Chrissa A. Dwyer, Donald P. Pizzo, Jaidev Bapat, Katrin Soldau, Ryotaro Ogawa, Mckenzie B. Riley, K. Peter R. Nilsson, Allison Kraus, Byron Caughey, Jeffrey J. Iliff, David R. Vera, Jeffrey D. Esko, Christina J. Sigurdson

**Affiliations:** 1 Department of Pathology, UC San Diego, La Jolla, California, United States of America; 2 Department of Cellular and Molecular Medicine, UC San Diego, La Jolla, California, United States of America; 3 Department of Radiology, UC San Diego, La Jolla, California, United States of America; 4 Laboratory of Persistent Viral Diseases, Rocky Mountain Laboratories, NIAID, NIH, Hamilton, Montana, United States of America; 5 Department of Pathology, Case Western Reserve University, Cleveland, Ohio, United States of America; 6 Department of Neurology, University of Alabama, Birmingham, Alabama, United States of America; 7 Department of Physics, Chemistry, and Biology, Linköping University, Linköping, Sweden; 8 VISN 20 NW Mental Illness Research, Education and Clinical Center, VA Puget Sound Health Care System, Seattle, Washington, United States of America; 9 Department of Psychiatry and Behavioral Science, Department of Neurology, University of Washington School of Medicine, Seattle, Washington, United States of America; 10 Department of Medicine, UC San Diego, La Jolla, California, United States of America; 11 Department of Pathology, Microbiology, and Immunology, UC Davis, Davis, California, United States of America; Creighton University, UNITED STATES

## Abstract

Select prion diseases are characterized by widespread cerebral plaque-like deposits of amyloid fibrils enriched in heparan sulfate (HS), a abundant extracellular matrix component. HS facilitates fibril formation *in vitro*, yet how HS impacts fibrillar plaque growth within the brain is unclear. Here we found that prion-bound HS chains are highly sulfated, and that the sulfation is essential for accelerating prion conversion *in vitro*. Using conditional knockout mice to deplete the HS sulfation enzyme, Ndst1 (N-deacetylase / N-sulfotransferase) from neurons or astrocytes, we investigated how reducing HS sulfation impacts survival and prion aggregate distribution during a prion infection. Neuronal Ndst1-depleted mice survived longer and showed fewer and smaller parenchymal plaques, shorter fibrils, and increased vascular amyloid, consistent with enhanced aggregate transit toward perivascular drainage channels. The prolonged survival was strain-dependent, affecting mice infected with extracellular, plaque-forming, but not membrane bound, prions. Live PET imaging revealed rapid clearance of recombinant prion protein monomers into the CSF of neuronal *Ndst1*- deficient mice, neuronal, further suggesting that HS sulfate groups hinder transit of extracellular prion protein monomers. Our results directly show how a host cofactor slows the spread of prion protein through the extracellular space and identify an enzyme to target to facilitate aggregate clearance.

## Author summary

Prions cause a rapidly progressive neurologic disease and death with no curative treatment available. Prion aggregates accumulate exponentially in the brain of affected individuals triggering neuronal loss and neuroinflammation, yet the molecules that facilitate prion protein aggregation are largely unknown. We have found that prions in the brain preferentially bind to a highly sulfated endogenous polysaccharide, known as heparan sulfate (HS). Here we use genetically modified mice that express poorly sulfated, neuron-derived HS, and infect mice with different prions strains. We find that mice infected with a plaque-forming prion strain show prolonged survival and fewer plaques compared to controls. We also found that recombinant prion protein was efficiently transported within the interstitial fluid of mice having poorly sulfated HS, suggesting more efficient clearance from the brain. Our study provides insight into how HS retains prion aggregates in the brain to accelerate disease and indicates a specific HS biosynthetic enzyme to target to enhance protein clearance.

## Introduction

The spread of aberrant protein aggregates through the brain is a key pathogenic mechanism in Alzheimer’s, Parkinson’s, and prion disease [1–6]. A potential mechanism for aggregate spread is by bulk transport within the extracellular space (ECS) [7–11]. The ECS harbors a dynamic reservoir of interstitial fluid (ISF) that flows toward perivascular clearance pathways [11]. Evidence suggests that bulk ISF flow is enhanced during sleep, fostering the efflux of metabolic waste and proteins, such as amyloid-β (Aβ) [12–14]. Thus, alterations in the structure or composition of the extracellular matrix (ECM) or perivascular clearance pathways during aging or neurodegenerative disease could hinder the clearance of peptides and oligomers [15–17].

Prion diseases are rapidly progressive neurodegenerative disorders in which prion protein aggregates (PrP^Sc^) deposit on cell membranes or as plaques embedded in the brain ECM, depending on the prion conformation [18–21]. Similar to Aβ plaques, PrP^Sc^ plaques are highly enriched in HS, a prominent component of the ECM [22–25]. Tissue HS proteoglycans (HSPGs) consist of long carbohydrate chains (40–300 alternating residues of glucuronic acid and glucosamine) attached to one or more protein cores [26,27]. A large body of evidence implicates HS in enhancing fibril formation and in the endocytosis of protein aggregates *in vitro* [28–32]. Moreover, polyanions administered intraventricularly increase survival time in experimental rodent models and in patients, potentially by blocking prion binding to endogenous HS [33–44]. Additionally, transgenic expression of mammalian heparanase, an enzyme that degrades HS, was reported to delay prion disease onset and progression [45].

HS chains bind and concentrate proteins, such as growth factors and cytokines, and the level and pattern of sulfation determine the binding affinity [26,27]. The enzyme, Ndst1, catalyzes the deacetylation and sulfation of HS chains at position N. Here, we show that brain-derived prions selectively bind highly sulfated HS chains. To understand how sulfation modifies prion aggregate spread and disease progression, we used conditional *Ndst1* knock-out mice to reduce the sulfation of either neuron or astrocyte generated HS, and challenged mice with diverse prion strains. We found that mice with reduced neuronal HS sulfation survived longer when infected with extracellular plaque-forming prions. The PrP^Sc^ conformational properties were largely unaltered except for a reduction in size, as fibrils were shorter and aggregates were more soluble. Additionally, fewer parenchymal plaques had formed, while vascular fibrils accumulated in the meninges. Finally, in positron emission tomography (PET) scan studies, radiolabeled soluble recombinant PrP (recPrP) rapidly diffused through the brain of mice expressing poorly sulfated neuronal HS, leading to increased PrP^C^ efflux into the spinal cord. Together, our studies identify how reducing HS sulfation increases PrP^Sc^ solubility, accelerates PrP clearance, reduces parenchymal plaque burden, and extends survival, demonstrating NDST1 as a potential therapeutic target.

## Results

### Highly sulfated HS binds PrP^Sc^

**Fig 1:**
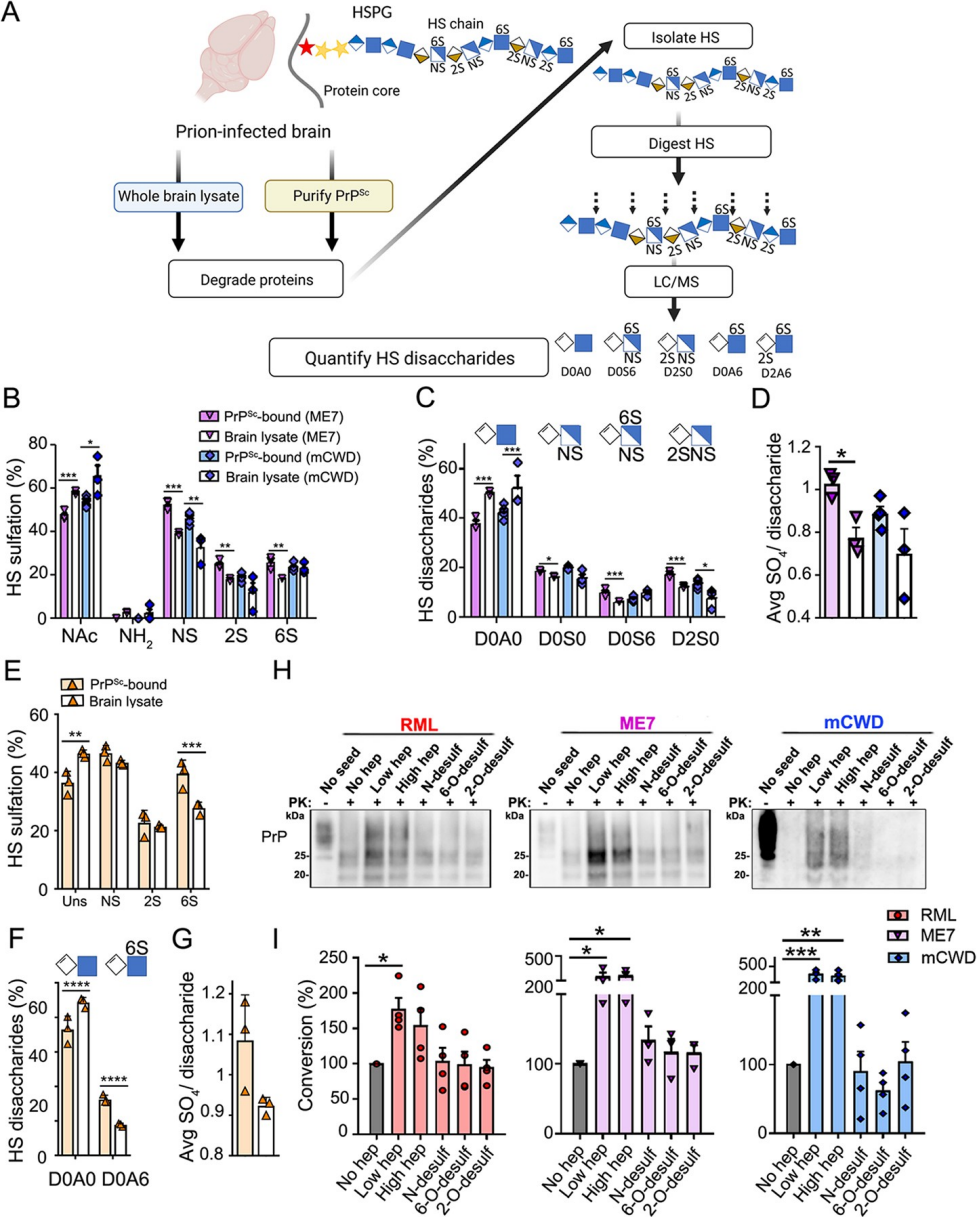
Mouse and human PrP^Sc^ bind highly sulfated HS. (A) Schematic of HS purification from brain lysate or purified PrP^Sc^ for mass spectrometry analysis (a subset of the latter samples was previously published [46,79]). (B) Quantification of unsulfated (NAc and NH_2_) and sulfated (NS, 2S, and 6S) HS, (C) individual HS disaccharides, and (D) average sulfation per disaccharide of HS bound to PrP^Sc^ as compared to brain lysates (same brain) (*n* = 3 mice per strain). (E) Quantification of unsulfated and sulfated HS from sCJD-affected brain, (F) individual HS disaccharides, and (G) average sulfation per disaccharide of HS bound to PrP^Sc^ as compared to brain lysates (same brain). *N* = 3 per group (occipital cortex). (H) Representative western blots and (I) quantification of PrP^Sc^-seeded PMCA in the presence or absence of heparin or heparin desulfated at positions N-, 6-O, or 2-O (No seed: no PrP^Sc^, No hep: no heparin, Low hep: 225 μg/ml of heparin, High hep: 2.225 mg/ml of heparin, N-, 6-O-, or 2-O-desulfated heparin: 225 μg/ml). N = 3–4 experimental replicates. Note for panels C and F, other disaccharides were not significantly different (shown in S1–S3 Tables). *P< 0.05, **P< 0.01, ***P< 0.005 and ****P< 0.001, two-way ANOVA with Bonferroni’s post test comparing within a single strain (panels B, C, E, and F), unpaired two-tailed t-test with Bonferroni’s post test (panels D and G), and one-way ANOVA with Tukey’s post test (panel I). NAc: N-acetylglucosamine (GlcNAc); NH_2_: glucosamine (GlcNH_2_); NS: N-sulfated glucosamine (GlcNS); 2S: 2-O-sulfated glucuronic or iduronic acids (2-O-S); 6S: 6-O-sulfated glucosamine (6-O-S); PK: proteinase K. Panel A created with BioRender.com.

To first establish the level and composition of HS molecules bound to PrP^Sc^ as compared to whole brain lysate, we performed liquid chromatography—mass spectrometry (LC/MS) analysis on whole brain lysate, comparing to HS molecules bound to PrP^Sc^ from the same brain [the latter previously reported [46]] (Fig 1A). For two prion strains (ME7 and mCWD), HS bound to PrP^Sc^ was 7–9% more sulfated (N-, 2-O-, and 6-O-sulfated) than HS in the brain lysate (Fig 1B–1D), indicating that more highly sulfated HS selectively binds PrP^Sc^. Prion-bound HS was particularly enriched in D2S0, a disulfated disaccharide (N- and 2-O-sulfated) (Fig 1C and S1 and S2 Tables). We repeated this experiment with cerebral cortex from human sporadic prion disease (sCJD)-affected brain. Consistent with PrP^Sc^ from mouse brain, the HS bound to PrP^Sc^ was again more highly sulfated [prion-bound: 63% sulfation versus brain lysate: 53%] (Fig 1E–1G). Additionally, human PrP^Sc^-bound HS was enriched in the disaccharide, D0A6 (Figs 1F and S1 and S3 and S4 Tables).

Heparin is more sulfated than HS and promotes the fibril formation of PrP^Sc^, Aβ, tau, and α-synuclein *in vitro* [47–52]. To determine how the HS sulfate position impacts the interaction with PrP, we tested a library of selectively desulfated heparin molecules lacking the N-, 6-O-, or 2-O-sulfate group in a prion conversion assay known as protein misfolding cyclic amplification (PMCA) [53]. A PrP^C^-expressing cell lysate was seeded with mouse prions (three strains) with and without heparin. Heparin increased prion conversion by 60–350% (Fig 1H–1I). Notably, eliminating any sulfate group, NS, 6S, or 2S, reduced conversion to baseline, indicating that sulfation at all three positions was necessary to accelerate prion conversion.

HS binds ligands through electrostatic interactions between the anionic sulfate groups and cationic amino acids in the ligand [26]. To identify the HS binding domain on PrP^C^, we performed alanine substitutions in three positively charged domains and an asparagine-rich domain (S2A Fig). Segments 23–27 and 101–110 were critical to PrP^C^–HS binding, as lysine and arginine substitutions at either site reduced binding by nearly 3-fold, while asparagine to alanine substitutions (segment 171–174) did not impact binding (S2B–S2D Fig). Together, these results suggest that the electrostatic interaction between the N-terminal lysine and arginine residues of PrP^C^ and the HS sulfate groups are essential for PrP^C^—HS binding.

### Depleting neuronal HS sulfate groups reduces parenchymal prion plaque number and prolongs survival

Given the findings that PrP^Sc^ selectively binds highly sulfated HS and that the sulfate groups enhance PrP^C^ conversion *in vitro*, we predicted that reducing HS sulfation would slow PrP conversion kinetics. To determine how less sulfated HS in the brain impacts PrP^Sc^ conformation and disease progression *in vivo*, we used *Ndst1*^*f/f*^ mice to conditionally delete *Ndst1* from neurons by crossing mice to a neuron-specific Cre-recombinase line driven by the *synapsin1* promoter (*SynCre*) (S3A Fig). To validate the mouse line, an LC/MS analysis of the *Ndst1*^*f/f*^*SynCre*^*+/-*^ and *SynCre*^*-/-*^ (subsequently referred to as *SynCre+* and *SynCre-*) brain was performed and revealed that the *SynCre+* brain had less sulfated HS (approximately 33% less sulfation per disaccharide) and fewer N-, 2-O- and 6-O-sulfated disaccharides, particularly D2S0 and D2S6 disaccharide units (S3B–S3C Fig and S5 Table). The *SynCre+* mice showed no clinical phenotype, brain lesions, nor change in PrP^C^ expression in the brain (S3D–S3H Fig), but showed lower microglial reactivity in cortex, thalamus, and hippocampus as compared to *SynCre-* mice (S3E–S3F Fig).

Sulfated HS enhances the uptake of prion fibrils in cultured cells [28,54] and may impact the conversion and spread of GPI-anchored and GPI-anchorless prions. Therefore, the *SynCre*+ and *SynCre-* mice were inoculated with prions that form either diffuse (RML; GPI-anchored) or diffuse and small plaque-like deposits (ME7; GPI- anchored and anchorless [46]). Mice infected with RML prions showed no difference in survival time [*SynCre-*: 158 ± 3 and *SynCre+*: 168 ± 4 days post-inoculation (dpi) (mean ± standard error)] or brain lesions (S4A–S4C Fig), indicating that neuronal HS (nHS) sulfation level had no impact on the replication or spread of a GPI-anchored, oligomeric prion strain. In contrast, *SynCre*+ mice infected with ME7 prions showed a significantly prolonged survival [*SynCre-*: 172 ± 6 and *SynCre+*: 199 ± 3 dpi] (S4A Fig). Notably, the prolonged survival was not associated with differences in the spongiosis, glial activation, or PrP^Sc^ level or distribution in brain, nor in the PrP^Sc^ biochemical properties, including electrophoretic mobility and glycoprofile (S4B–S4H Fig), suggesting that reducing nHS sulfation primarily affected the kinetics of prion conversion and spread.

**Fig 2:**
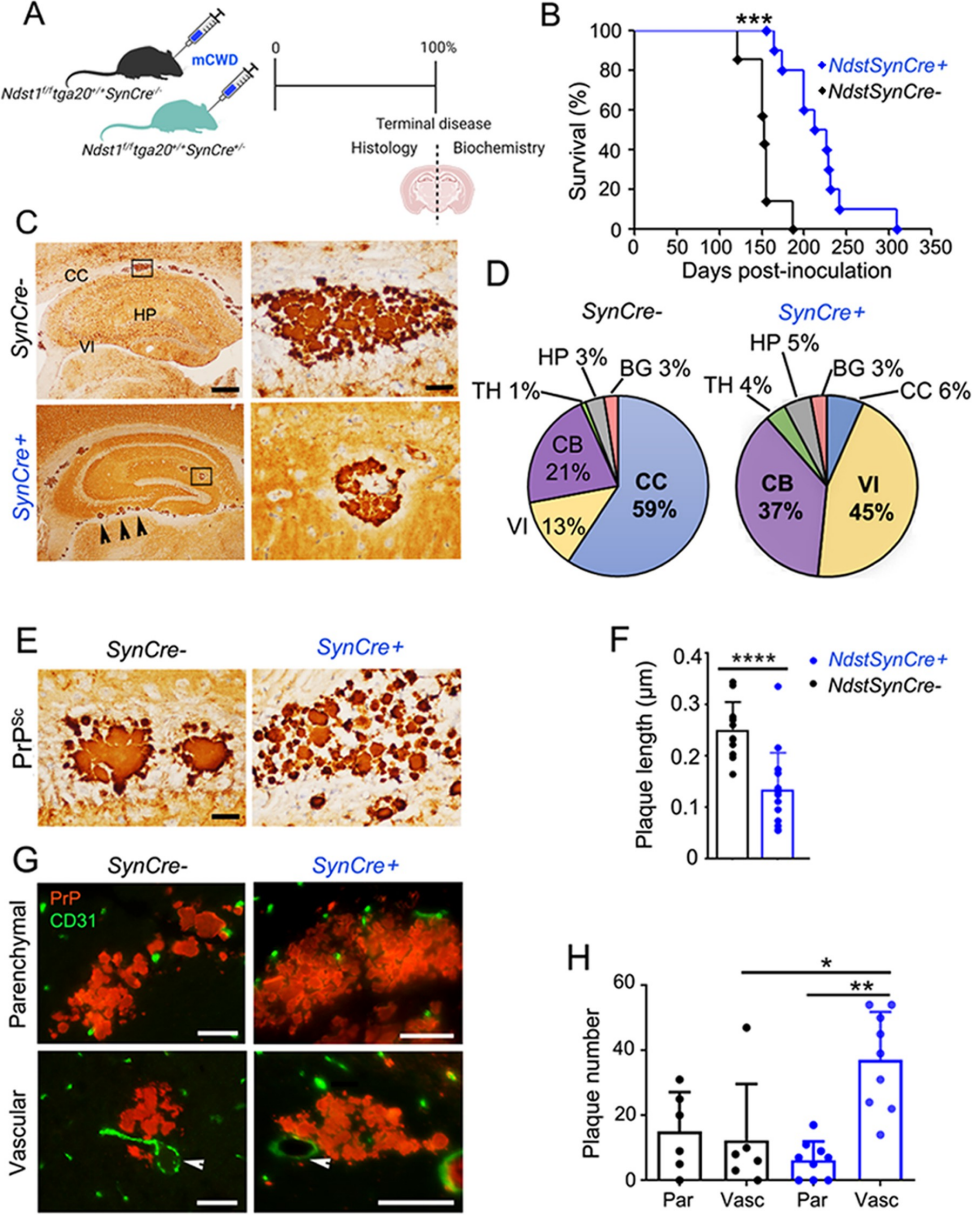
Reducing neuronal HS sulfation prolongs survival and lessens plaque load in prion-affected mice. (A) Schematic illustrates mCWD prion inoculation and tissue collection. (B) Survival curves for mCWD-infected *SynCre-* (*n* = 10) and *SynCre+* mice (*n* = 15). (C) Representative images reveal mCWD prion plaques in the corpus callosum (CC) of *SynCre-* mice and within vessels of the velum interpositum (VI) and hippocampus (HP) of *SynCre+* mice. Higher magnification depicted in right panels. Scale bars represent 500 μm (left) and 50 μm (right). (D) Pie charts show the plaque distribution in brain. VI and cerebellar (CB) aggregates were primarily vascular (meninges) (*n* = 6 *SynCre-* and 9 *SynCre+* mice). S6 Table indicates the plaque number in each brain area. (E) Representative images of PrP^Sc^ immunolabelled plaques in CC. Scale bar = 50 μm. (F) Quantification of the plaque length (CC) (*n* = 33 and 38 plaques in *SynCre-* and *SynCre+*, respectively) (*n* = 4 mice per genotype). (G) Representative example of dual immunolabelled mCWD-infected brain sections for PrP^Sc^ and endothelial cells (CD31) (parenchymal plaques: corpus callosum; *SynCre-* vascular plaque: basal ganglia; *SynCre+* vascular plaque: thalamus). Scale bar represents 50 μm. (H) Quantification of parenchymal (par) and vascular (vasc) plaques in cerebral cortex, septum, corpus callosum, hippocampus, thalamus, cerebral peduncle, hypothalamus, cerebellar peduncle, velum interpositum, cerebellum, and medulla. *N* = 6 *SynCre-* and 9 *SynCre+* mice. *P< 0.05, **P< 0.01, ***P< 0.005, and ****P< 0.001, Log-rank (Mantel-Cox) test (panel B), unpaired two-tailed t-test with Bonferroni’s post test (panel F), and two-way ANOVA with Bonferroni’s post test (panel H). TH thalamus, BG: basal ganglia, and CT: cerebral cortex. Panel A created with BioRender.com.

Since prion fibrils bind 4- to 10-fold more HS than subfibrillar aggregates (μg HS / μg PrP^Sc^) [46], we predicted that reducing HS sulfation would substantially prolong survival in mice infected with the plaque-forming, GPI-anchorless prion strain, mCWD [55]. Because the disease course for mCWD prions in WT mice is more than 550 days, we crossed the *Ndst1*^*f/f*^*SynCre*^*+/-*^ mice to *tga20*^*+/+*^ mice, which overexpress PrP^C^ (4- to 6-fold) and show accelerated disease progression [55,56]. Strikingly, mCWD-infected *Ndst1*^*f/f*^*tga20*^*+/+*^*SynCre*^*+/-*^ mice (hereafter noted as *SynCre+*) displayed a markedly prolonged survival, approximately 40% longer than the *SynCre-* mice [*SynCre-*: 154 ± 6 and *SynCre+*: 206 ± 10 dpi] (Fig 2A and 2B).

Decreasing nHS sulfation had a pronounced effect on the prion plaque distribution in the *SynCre+* brain. While most *SynCre-* mice (83%; n = 5/6 mice) accumulated parenchymal plaques in the corpus callosum (CC), only one-third of the *SynCre+* developed plaques in the CC (33%; n = 3/9 mice) (Fig 2C and 2D), which were smaller and multicentric (clusters of small plaques) (Fig 2E and 2F). The *SynCre+* brain also showed an increase in vascular amyloid (*SynCre-*: 12 ± 2 versus *SynCre+*: 37 ± 2 amyloid-laden vessels), particularly within the velum interpositum and cerebellar meninges (Figs 2C, 2D, 2G, 2H, and S5A–S5B and S6 Table), suggestive of enhanced prion transit toward perivascular drainage pathways. There were no differences in the astrocytic or microglial response to aggregates (S5C–S5D Fig). Additionally, HS was still detectable in plaques (S5E Fig), and there was no change in plaque morphology in situ by electron microscopy (S5F Fig). Finally, fluorescent lifetime decay (FLIM) of a prion-bound fluorescent probe, heptameric formic thiophene acetic acid (h-FTAA), revealed no differences, suggesting no change in the PrP^Sc^ conformational properties (S6A Fig). Thus, reducing HS sulfation prolonged survival and reduced parenchymal plaques and plaque size in the brain, without detectably altering PrP^Sc^ secondary or tertiary conformation.

**Fig 3:**
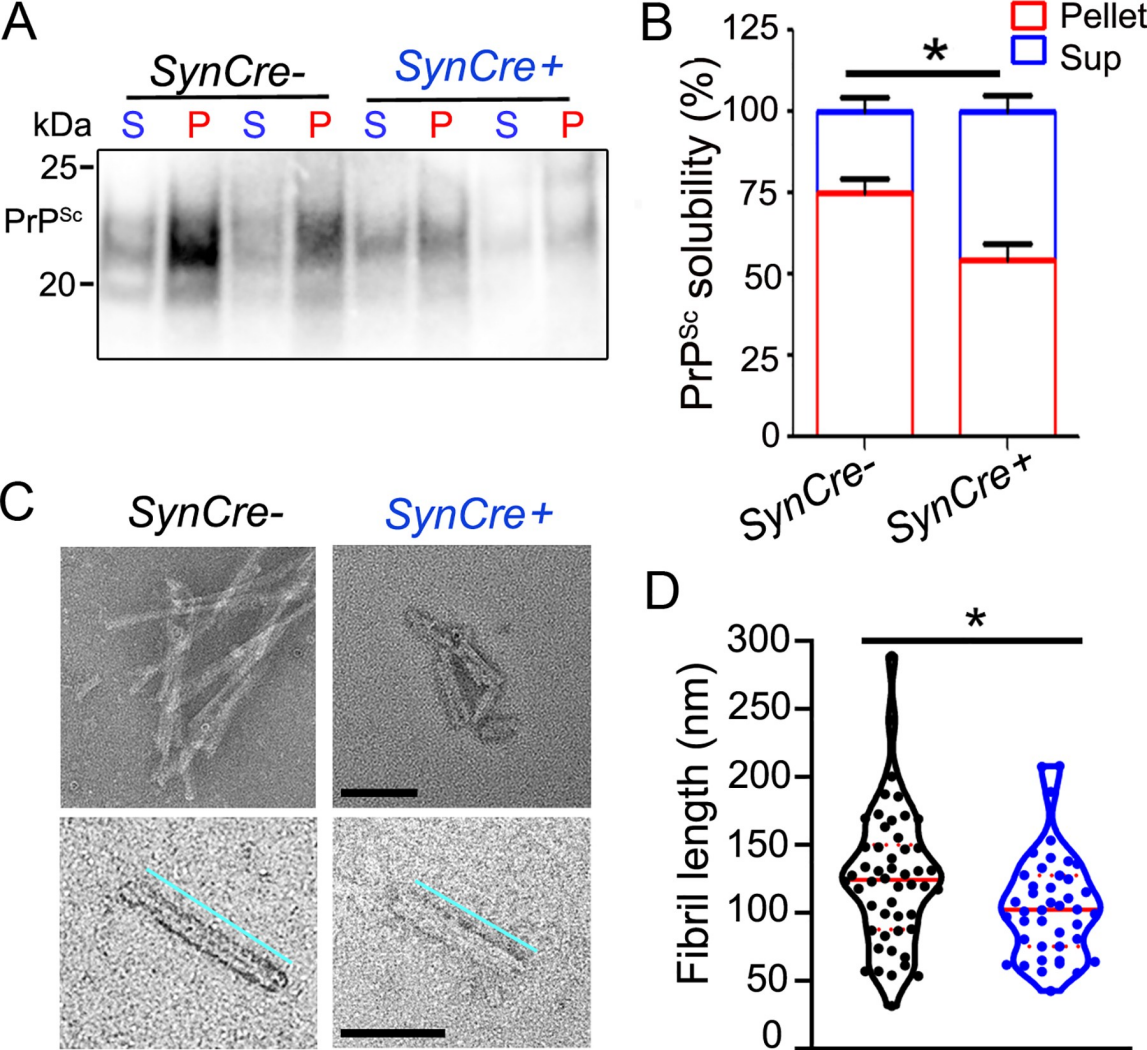
Reducing neuronal HS sulfation increases mCWD solubility and decreases fibril length. (A) Representative western blot of mCWD aggregates after PK digestion and centrifugation over an Optiprep layer (P = pellet, S = supernatant). (B) Quantification of PrP^Sc^ in pellet and supernatant fractions. *N* = 6 samples per genotype. (C) Representative electron microscopy images of purified PrP^Sc^ fibrils from mCWD-infected mice. Scale bars represent 100 nm. Fibril measurements (bottom panel) are 193 nm (*SynCre-*) and 144 nm (*SynCre+*). (D) Quantification of the fibril lengths. *N* = 49 and 42 fibrils measured from *SynCre-* and *SynCre+* brains, respectively (*n* = 6 mice per genotype*)*. *P< 0.05, unpaired two-tailed t-test with Bonferroni’s post test (panels B and D).

To further test PrP^Sc^ fibril conformation using biochemical analyses, we measured the biochemical properties of PrP^Sc^, including the electrophoretic mobility of the proteinase K (PK)-resistant core, glycoprofile, stability in chaotropes, and aggregate solubility (Figs 3A, 3B, and S6B–S6E). Notably the only difference was in PrP^Sc^ solubility, in which there was an increase (25%) in the *SynCre*^*+*^ brains (Fig 3A and 3B) suggesting that the aggregate population was generally smaller. To further assess aggregate size, we next purified prion fibrils from brain homogenate and measured the length of isolated fibrils by electron microscopy. We found that fibrils were approximately 20% shorter in the *SynCre+* brains (Fig 3C and 3D) (n = 49 and 42 extracted fibrils measured from *SynCre*- and *SynCre*+ brains, respectively). Taken together, these data suggest that neuron-derived sulfated HS enhances PrP^Sc^ fibril elongation.

The increased PrP^Sc^ clearance into the cerebrospinal fluid (CSF) may lead to enhanced deposition in the spinal cord. Thus, we next compared the PrP^Sc^ seeding activity in the spinal cord from *SynCre*- and *SynCre*+ using real-time quaking-induced conversion (RT-QuIC). However, there were no differences in the proportion of mice with seeding activity in the spinal cord (*SynCre*-: 7 of 11 mice, *SynCre*+: 14 of 16 mice; Fisher’s exact test; *p* = 0.19) (S7 Table), suggesting that reducing nHS sulfation does not lead to an increased incidence of PrP^Sc^ deposition in the spinal cord.

### Decreasing neuronal HS sulfation enhances recPrP clearance into the spinal cord

**Fig 4:**
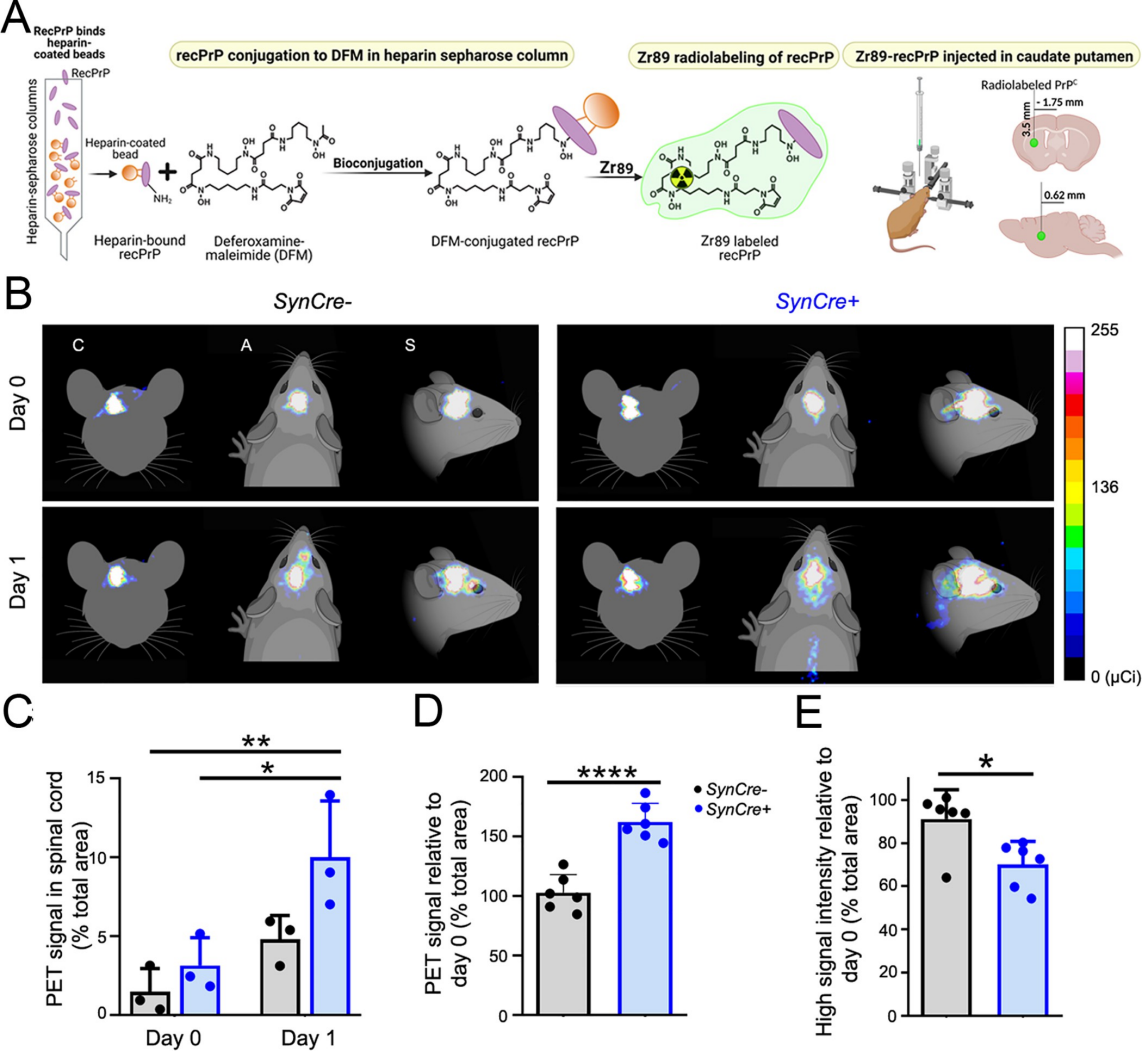
Rapid transit of Zr89-recPrP through the brain and spinal cord of live *Ndst1*^*f/f*^*tga20*^*+/+*^*SynCre*+ mice. (A) Schematic shows the conjugation, radiolabeling, and stereotaxic injection of recPrP into anesthetized *Ndst1*^*f/f*^*tga20*^*+/+*^*SynCre*+ and *SynCre-* mice. (B) Representative PET scan images (coronal (C), axial (A) and sagittal (S) sections) of Zr89-recPrP in *SynCre-* and *SynCre+* mice immediately after injection into the caudate putamen (day 0) and 20 hours later (day 1) in *n* = 6 animals per genotype (two experiments). Graphs show (C) radioactive recPrP in the spinal cord at day 0 and day 1 relative to the total PET signal area, as well as (D) the total PET signal area at day 1 relative to day 0 (sagittal, brain and spinal cord) and (E) the high PET intensity signal area (signal > 100 μCi) at day 1 relative to day 0. Panel C shows only one experiment. *P< 0.05, **P< 0.01, and ****P< 0.001, two-way ANOVA with Bonferroni’s post-test (panel C), and unpaired two-tailed t-test with Bonferroni’s post test (panels D and E). Panels A and B created with BioRender.com.

Given that i) HS is a significant component of the brain ECM and ii) decreasing nHS sulfation reduces parenchymal plaque load and prolongs survival in prion-infected mice (Figs 2B–2H and S5A), we reasoned that HS may entrap PrP in the ECM, hindering clearance by ISF bulk flow. To investigate how reducing HS sulfation affects soluble PrP^C^ transit through the ISF, we used PET to track radiolabeled recPrP in real time (Fig 4A). RecPrP was radiolabelled with zirconium-89 (Zr89) (recPrP-Zr89) (radiolabel confirmed in S7A Fig). Zr89-labelled recPrP was stereotaxically injected into the left caudate putamen of *Ndst1*^*f/f*^*tga20*^*+/+*^*SynCre*^*+/-*^ and *SynCre*^*-/-*^ mice in two experiments. Mice were imaged by PET immediately after injection [day 0 (D0)] and at 20 hours post-injection (D1) (Figs 4B and S7).

PET imaging immediately after injection revealed intense radioactive signal (more than 100 μCi) similarly concentrated at the injection site in *SynCre*- and *SynCre*+ brains, with less than 5% in the spinal cord (Figs 4B–4D and S7B–S7C). Strikingly, at D1, the total recPrP-Zr89 radiolabeled area on a sagittal measurement had increased by approximately 68% in the *SynCre*+ brain, in contrast to minimal change (9%) in the *SynCre*- brain (Figs 4B, 4D, and S7D). Moreover, there was a pronounced decrease (30%) in the intense radioactive core area (more than 100 μCi) in the *SynCre*+ mice (9% in *Cre*-) (Fig 4E), suggestive of enhanced recPrP-Zr89 diffusion from the injection site. Additionally, when comparing the radioactive signal in the spinal cord over time, there was an approximately 14-fold difference in the signal change observed in the *SynCre*+ as compared to *SynCre-* mice (20% versus 287% increase in spinal cord signal at D1 compared to D0 in *SynCre*- and *SynCre*+ mice, respectively) (Fig 4C), indicating that more recPrP-Zr89 had spread from the brain to the spinal cord in the *SynCre*+ mice. Collectively these studies suggest that decreasing HS sulfation accelerates PrP^C^ clearance.

### Altering astrocytic HS sulfation does not impact prion disease progression

Since the HS in the CNS is secreted by neurons and glial cells [57–59], we next tested how reducing astrocyte-derived HS sulfation would affect prion conversion *in vivo*. *Ndst1*^*f/f*^ mice were crossed with mice expressing *GFAPCre* (glial fibrillary acidic protein), as well as to *tga20* mice for mCWD inoculation. Brain lysates from uninfected aged *Ndst1*^*f/f*^*GFAPCre*+ (hereafter *GFAPCre*+) mice showed modest changes in HS composition (S8A-S8C Fig and S8 Table). Mice showed no change in PrP^C^ expression level, and no clinical or neuropathologic phenotype (S8D-S8F Fig). *GFAPCre*+ and *GFAPCre*- littermates were intracerebrally inoculated with RML, ME7, or mCWD prions. Interestingly, the prion-infected *GFAPCre*+ and *Cre*- showed similar survival times [Cre+ and Cre-: RML: 160 ± 5 versus 162 ± 1 dpi, ME7: 182 ± 4 versus 191 ± 4 dpi, mCWD: 140 ± 5 versus 133 ± 5 dpi, respectively]. Additionally, there were no differences in brain lesions (PrP^Sc^ distribution, vacuolation, or astrocytic gliosis) or biochemical properties of PrP^Sc^ (S8G–S8Q Fig). These results indicate that reducing astrocytic HS sulfation had no detectable impact on prion disease progression, pathologic phenotype, or PrP^Sc^ biochemical properties for mice infected with three distinct strains of prions, in striking contrast to the effect of reducing neuronal HS sulfation.

In summary, our study demonstrates a previously unrecognized role for highly sulfated HS chains in advancing prion disease progression. We found evidence that HS and PrP bind by electrostatic interaction, with PrP^Sc^ and sulfated HS engaging within the brain parenchyma, enhancing fibril elongation, parenchymal plaque formation, and markedly accelerating disease. Interestingly, this effect was HS cell source and prion strain dependent, as reducing neuronal HS, but not astrocytic HS, prolonged survival, and only impacted strains with a GPI-anchorless component. Finally, we systematically identified highly sulfated HS molecules concentrated within prion aggregates in mouse and human brain by LC/MS, further supporting NDST1 as a therapeutic target.

## Discussion

PrP^Sc^ and Aβ plaques are enriched in HS [23,25,46,60], yet whether HS facilitates fibril formation *in vivo* has been unclear. Here we establish that neuron-generated HS accelerates prion propagation in a strain-dependent manner. We show the importance and specificity of HS sulfate groups in binding PrP, as the HS molecules enriched within prion aggregates were more highly 6-O-, 2-O-, or N-sulfated compared to brain lysate, and sulfation was key to amplifying prion conversion. Notably, depleting nHS sulfation reduced parenchymal plaque load, increased meningeal amyloid, and dramatically improved survival in mice infected with mCWD, collectively supporting a model of enhanced prion clearance in mice with poorly sulfated HS. Live imaging studies supported this model, revealing accelerated efflux of recPrP monomer into the spinal cord. Thus, we propose that reducing HS sulfation is sufficient to impair HS—PrP^Sc^ interaction and augment extracellular PrP egress into the CSF, prolonging the survival of mice with prion disease.

Previous studies report heparin and HS promote fibril formation *in vitro* [38,50], and prion-infected mice and variant CJD (vCJD) patients treated with HS mimetics survive longer [33–35, 39–44]. Using genetic models, we and others recently found that shortening HS chains reduces plaque load and extends survival or improves behavior in prion-infected and Alzheimer’s mouse models, respectively [46,61], implicating HS in both prion and Aβ plaque formation *in vivo*. Our studies now demonstrate the highly sulfated nature of HS bound to prions using LC/MS, and the feasibility of manipulating HS biosynthetic pathways to enhance PrP clearance and increase resistance to extracellular plaque formation. Notably, the few parenchymal plaques present in mice expressing less sulfated nHS were small and the isolated fibrils shorter, together providing strong evidence to support that sulfated HS recruits prion aggregates, facilitating fibril elongation and plaque formation in the brain parenchyma. Whether other reported co-factors, such as lipids or nucleic acids [62–64], also promote PrP^C^ to PrP^Sc^ conversion *in vivo* is unclear and would be important to address in future studies. Nevertheless, manipulating HS polymerase or sulfotransferase expression to reduce HS length or sulfation, respectively, may be a viable therapeutic strategy to promote the clearance of GPI-anchorless prions, Aβ, and other extracellular aggregates.

Reducing nHS sulfation did not affect the disease progression of *Ndst1*^*f/f*^*SynCre*^*+/-*^ mice infected with GPI-anchored prions (RML), despite PrP^C^ and PrP^Sc^ being embedded within an extensive meshwork of cell surface HSPGs. *In vitro*, HS reportedly promotes the endocytosis of PrP^Sc^, as well as tau and α-synuclein [28–31,65,66], yet here reducing nHS sulfation did not detectably alter RML prion propagation, astrocyte reactivity, or neurotoxicity. Although we found that heparin binds with high affinity and promotes prion conversion *in vitro*, minimal HS was bound to GPI-anchored prions isolated from brain [46], suggesting that the PrP^Sc^ membrane location limits access to HS, and HS may not alter neurotoxicity *in vivo*. Given that GPI-anchored prions concentrate within lipid rafts, the membrane curvature or phosphate head groups, lipid raft size, or HS location and spatial orientation may hinder HS–PrP^C^ or HS–PrP^Sc^ binding and constrain fibril elongation for membrane-bound prions, abrogating any major scaffolding effect by HS [67]. In contrast, extracellular PrP^Sc^ is unconstrained in the parenchyma and may more readily bind extracellular HS chains, consistent with our previous finding of abundant HS bound to three strains of GPI-anchorless PrP^Sc^ [46]. That said, the GPI-anchorless strains used here were limited, and we cannot exclude that HS promotes the endocytosis, neuronal toxicity, or propagation of other prion conformers, as prions propagating in different brain regions may access HS chains with a sulfation code better suited for binding.

Notably, reducing nHS sulfation impacted disease progression, however reducing astrocytic HS sulfation had no effect. Although both neurons and astrocytes produce and secrete HS [57,58,68], PrP may selectively bind nHS due to the level and pattern of HS sulfation. Alternatively, considering that neuronal PrP^C^ is essential and sufficient for PrP-linked toxicity [69], it is conceivable that membrane-bound HSPGs such as syndecans or glypicans, facilitate extracellular PrP^Sc^ (GPI-anchorless) binding to a neuronal surface receptor complex in trans, potentiating neurotoxic signaling pathways. In this case, reducing nHS sulfation may decrease PrP^Sc^ interactions with neuronal surface receptors. Future studies may further elucidate the mechanism underlying the prolonged survival.

Our studies support a broader role of HS and the ECM in retaining proteins and promoting aggregation in the aging brain, with implications beyond prion disease. Given that HS also binds lipids and chemokines, this work suggests that increases in HS levels or sulfation may slow protein efflux and increase neuroinflammation, particularly in the presence of protein aggregates. Similar to PrP, the N-terminus of Aβ also binds to HS [70] and HS sulfate groups are required for Aβ binding [71]. Given the similarities of our prion disease model to AD, future studies to (i) characterize how HS levels and composition change with age and disease in the human brain, (ii) determine whether HS enhances neuroinflammation during aging, (iii) define the structure of HS concentrated within Aβ plaques, and (iv) enhance the clearance of Aβ in AD models would be of high priority.

We demonstrate a significant increase in the lifespan of nHS-depleted mice infected with extracellular, plaque-forming prions. This finding suggests that HS biosynthetic enzymes, such as Ndst1, may be therapeutic targets for select neurodegenerative diseases in which extracellular amyloid plaques develop, including familial prion disease [Gerstmann-Sträussler-Scheinker disease (GSS)], vCJD, as well as Alzheimer’s disease. Reducing HS sulfation in early disease would be expected to enhance aggregate transport toward perivascular clearance pathways. However, a caveat is a possible increase in meningeal vascular amyloid, as observed in prion-infected mice with reduced HS sulfation or with shortened HS chains [46]. Given that these results highlight the importance of HS sulfation in plaque formation, future work may define and inhibit PrP^Sc^ and Aβ binding to vessel-associated molecules to further promote protein aggregate clearance into the CSF.

In conclusion, our data provide evidence that PrP^Sc^ selectively binds highly sulfated neuronal HS, thereby identifying HS as the first integral *in vivo* co-factor in prion fibril formation and plaque assembly. Reducing HS sulfation decreased the parenchymal plaque burden and extended survival, suggesting that *in vivo*, PrP—HS electrostatic interactions are critical for binding. We also directly demonstrate how sulfated HS slows recPrP clearance from the ISF in real time, suggesting that GPI-anchorless PrP^C^ transits through the brain by bulk flow, and that clearance may be increased by manipulating HS chemical properties. Importantly, our data strongly support the pursuit of therapeutic strategies targeting HS biosynthetic enzymes to facilitate the clearance of extracellular protein aggregates.

## Materials and methods

### Ethics statement

All animal studies were approved by the Institutional Animal Care and Use Committee at University of California, San Diego (protocol S08037). Protocols were performed in strict accordance with good animal practices, as described in the Guide for the Use and Care of Laboratory Animals published by the National Institutes of Health.

### Prion transmission studies in mice

*Ndst1*^*f/f*^ mice [72] were bred to mice that express the Cre-recombinase under the neuron and astrocyte specific promoters, *synapsin1* and *glial fibrillary acidic protein* (*SynCre* and *GFAPCre*), respectively, and for mCWD studies, mice were bred to *tga20* mice that overexpress mouse PrP^C^ [56]. Homozygosity for the *Prnp transgene* was determined by quantitative real time PCR using the PureLink Genomic Purification Kit, and Taqman Master Mix, Copy Number Assay (*Prnp*) and Copy Number Reference Assay, mouse, *Tfrc* (Thermo Fisher Scientific). Mice were maintained under specific pathogen-free conditions on a 12:12 light/dark cycle.

Male and female *Ndst1*^*f/f*^*SynCre+ or GFAPCre+* and *Ndst1*^*f/f*^*tga20*^*+/+*^*SynCre+ or GFAPCre*+ (6–8 weeks old) and *Cre-* littermate control mice were anesthetized with ketamine and xylazine and inoculated into the left parietal cortex with 30 μl of 1% prion-infected brain homogenate prepared from terminally ill mice (n = 7–17 mice/group) or with 1% brain homogenate from uninfected WT mice (mock) (n = 3–4 mice/group). Prion-inoculated mice were monitored three times weekly for the development of terminal prion disease, including ataxia, hyperactivity, kyphosis, stiff tail, hind leg clasp, and hind leg paresis, and were euthanized at the onset of terminal disease. During necropsy, the brain was halved, and the left hemisphere was immediately fixed in formalin. Fixed brains were treated for 1 hour in 96% formic acid, post-fixed in formalin, cut into 2 mm transverse sections, and paraffin-embedded for histological analysis. A 2–3 mm transverse section was removed from the left hemisphere at the level of the hippocampus/thalamus, embedded in optimal cutting temperature (OCT) compound and immediately frozen on dry ice. The remaining brain tissue was frozen for biochemical studies. Survival time was calculated from the day of inoculation to the day of terminal clinical disease.

### Histopathology and immunohistochemical stains

Five-micron sections were cut onto positively charged silanized glass slides and stained with hematoxylin and eosin (HE), or immunostained using antibodies against PrP (SAF84, epitope in the globular domain at the amino acids 160–170 of the mouse PrP), astrocytes (glial fibrillary acidic protein, GFAP), and microglia (Iba1). GFAP (DAKO; 1:6000), Iba1 (Wako; 1:3000), and PrP^Sc^ (Cayman Chemical; 1:1200) immunohistochemical labelling was performed on an automated tissue immunostainer (Ventana Discovery Ultra, Ventana Medical Systems). Each epitope had independently optimized retrieval parameters to yield the maximal signal to noise ratio. For PrP^Sc^, slides were incubated in protease 2 for 20 minutes followed by antigen retrieval in CC1 (tris-based; pH 8.5; Ventana) for 64 minutes at 95°C. For GFAP only the protease P2 was used (Ventana) for 16 minutes. Iba1 retrieval consisted of CC1 for 40 minutes at 95°C. Following retrieval, antibodies were incubated on the tissue for 32 minutes at 37°C. The secondary antibody (HRP-coupled goat anti-rabbit or anti-mouse; OmniMap system; Ventana) was incubated on the sections for 12 minutes at 37°C. The primary antibody was visualized using DAB as a chromogen followed by hematoxylin as a counterstain. Slides were rinsed, dehydrated through alcohol and xylene and cover slipped.

For the PrP^Sc^ and CD31 (endothelial cells) dual immunolabelling, tissue sections were stained sequentially using anti-PrP SAF84 (1:150) and CD31 antibodies (Dianova; 1:150) using the tyramide signal amplification system (TSA; ThermoFisher). Slides were stained on a Ventana Discovery Ultra (Ventana Medical Systems, Tucson, AZ, USA). Antigen retrieval was performed using a slightly basic treatment solution (CC1; pH 8.5, Ventana) for 92 minutes at 95°C. Sections then were incubated in anti-PrP antibody for 32 minutes at 37°C, followed by anti-mouse-HRP (UltraMap Detection Kit, Ventana) and TSA-Alexa 594. The antibodies were denatured by treatment in a citric acid-based solution, pH 6 (CC2, Ventana) for 24 minutes at 95°C. Subsequently, the slides were incubated with anti-CD31 antibody (rat) for 32 minutes at 37°C followed by rabbit anti-rat (1:500; Jackson ImmunoResearch) and detected using the OmniMap system (Ventana) to fluorescently label CD31-expressing endothelial cells with TSA-Alexa 488. Parenchymal and CD31-positive vascular plaques were manually quantified in the cerebral cortex, thalamus, cerebral peduncle, hippocampus, septum, hypothalamus, cerebellar peduncle, corpus callosum, velum interpositum, cerebellum, and medulla by an investigator blinded to animal identification.

The PrP^Sc^ and HS dual immunolabelling was performed as previously described but with minor differences [46]. Briefly, tissue sections were deparaffinized, and epitope exposure was performed using formic acid followed by PK, prior to heating in a citrate buffer (pH 6). Sections were blocked and incubated with anti-PrP SAF-84 (1:400) antibody for 1 hour followed by IgG–CY3 (Jackson Immunolabs; 1:200) for 30 minutes. Sections were next incubated with anti-HS 10E4 antibody (AMS Bioscience; 1:300), anti-mouse IgM biotin (Jackson Immunolabs; 1:500) for 30 minutes, streptavidin-HRP (Jackson Immunoresearch; 1:2000) for 45 minutes and tyramide-Alexa488 (Invitrogen) for 10 minutes. Nuclei were labeled with DAPI, and slides were mounted with fluorescent mounting medium (ProLong Gold antifade reagent). As controls for the HS stain, a subset of duplicate slides was treated with 8 milliunits of heparin lyases I, II, and III for 1 hour prior to immunostaining. Isotype immunoglobulin controls, single sections immunostained for PrP^Sc^ or HS, and prion negative (uninfected) cases were also included.

### Lesion profile

Brain lesions from prion-infected mice were scored for the level of PrP^Sc^ immunological reactivity, spongiosis, and astrocytic gliosis on a scale of 0–3 (0 = not detectable, 1 = mild, 2 = moderate, 3 = severe) in 9 regions: (1) dorsal medulla, (2) cerebellum, (3) hypothalamus, (4) medial thalamus, (5) hippocampus, (6) septum, (7) medial cerebral cortex dorsal to hippocampus, (8) cerebral peduncle, and (9) cerebellar peduncle. A sum of the three scores resulted in the value obtained for the lesion profile for the individual animal in a specific brain area and was depicted in the ‘radar plots’. An investigator blinded to animal identification performed the histological analyses.

### Quantitative analysis of astrocytic and microglial inflammation

To measure astrocytic reactivity and microglial activation in *Ndst1*^*f/f*^*SynCre*, *Ndst1*^*f/f*^*tga20*^*+/+*^*SynCre*, *Ndst1*^*f/f*^*GFAPCre* and *Ndst1*^*f/f*^*tga20*^*+/+*^*GFAPCre* mice, slides containing cerebral cortex, corpus callosum, hippocampus, thalamus, hypothalamus, and cerebellum were imaged using the Olympus EX41 microscope with DP Controller. Images were converted to grayscale, and FIJI (an ImageJ based image processing software) was used to measure the total brain area and quantify astrocyte and microglia reactivity using the “Measure” function. Astrocyte and microglia were demarcated using the “Find the edges” function and particle analysis was used to measure the area occupied. The total area covered by astrocyte and microglia was divided by the total area for each brain region.

### Western blot and glycoprofile analyses

For ME7-infected WT mice, PrP^Sc^ was concentrated from 10% brain homogenate in phosphate buffered saline (PBS) (w/v) by performing sodium phosphotungstic acid (NaPTA) precipitation prior to western blotting [73]. Briefly, 20 μl of 10% brain homogenate in an equal volume of 4% sarkosyl in PBS was nuclease digested (benzonase, Sigma) followed by digestion with 20 μg/ml PK at 37°C for 30 minutes. After addition of 4% sodium phosphotungstic acid in 170 mM MgCl_2_ and protease inhibitors (Complete, Roche), extracts were incubated at 37°C for 30 minutes and centrifuged at 18,000 x g for 30 minutes at 25°C. Pellets were resuspended in 2% sarkosyl prior to electrophoresis and immunoblotting. Samples were electrophoresed in 10% Bis-Tris gel (Invitrogen) and transferred to nitrocellulose by wet blotting. Membranes were incubated with monoclonal antibody POM19 [discontinuous epitope at C-terminal domain, amino acids 201–225 of the mouse PrP [74]] followed by incubation with an HRP-conjugated IgG secondary antibody. The blots were developed using a chemiluminescent substrate (Supersignal West Dura ECL, ThermoFisher Scientific) and visualized on a Fuji LAS 4000 imager. Quantification of PrP^Sc^ glycoforms was performed using Multigauge V3 software (Fujifilm). For mCWD, 100 μl of 10% brain homogenate was concentrated using NaPTA as described above, except samples were digested with 100 μg/ml PK for 45 minutes.

### Conformation stability assay

To measure prion strain stability in guanidine hydrochloride (GdnHCl), 10% brain homogenates were denatured for 1 hour in increasing concentrations of GdnHCl from 0 to 6 M. Samples were then diluted with a Tris-based lysis buffer (10 mM Tris-HCl, 150 mM NaCl, 10 mM EDTA, 2% sarkosyl, pH 7.5) to 0.15 M GdnHCl and digested with PK at a ratio of 1:500 (1 μg PK: 500 μg total protein) for 1 hour at 37°C. The digestion was stopped with 2 mM phenylmethylsulfonyl fluoride (PMSF) and protease inhibitors (Complete, Roche) followed by centrifugation at 18,000 x g for 1 hour. Pellets were washed in 0.1 M NaHCO_3_ (pH 9.8) and centrifuged at 18,000 x g for 20 minutes. Pellets were then denatured in 6 M guanidine isothiocyanate, diluted with 0.1 M NaHCO_3_, and coated passively onto an ELISA plate. PrP^Sc^ was detected with biotinylated-POM1 antibody (epitope in the globular domain, amino acids 121–231 of the mouse PrP [74]), a streptavidin HRP-conjugated secondary antibody, and a chemiluminescent substrate. Stability was measured in a minimum of 3 independent experiments, comparing *Ndst1*^*f/f*^*tga20*^*+/+*^*SynCre+* with *SynCre*–mice (3–4 mice per group).

### PrP^Sc^ solubility assay

Brain homogenates were solubilized in 10% sarcosyl in PBS and digested with 50 μg/ml of PK (final concentration) at 37°C for 30 minutes. Protease inhibitors were added (Complete, Roche), and samples were layered over 15% Optiprep and centrifuged at 18,000 x g for 30 minutes at 4°C. Supernatants were removed and pellets were resuspended in PBS in a volume equivalent to the supernatant. Supernatant and pellet fractions were immunoblotted using anti-PrP antibody POM19. PrP^Sc^ signals were captured and quantified as described under Western blot (3–4 mice per group).

### Cell-lysate protein misfolding cyclic amplification (clPMCA)

The pcDNA3.1 vector (Invitrogen) with the mouse *Prnp* encoding the 3F4 epitope (109M, 112M human numbering) was used as a template for site-directed mutagenesis (QuikChange Site Directed Mutagenesis kit, Agilent). PrP-deficient RK13 cells (ATCC) were transfected with 5–10 μg of plasmid DNA encoding mutant PrP using lipofectamine 3000 (Invitrogen). At 24 hours post-transfection, cells were washed twice in PBS, harvested in 1 ml PBS, and centrifuged for 1 minute at 1000 x g. The pellet was resuspended in PMCA buffer (PBS containing 1% triton X-100, 150 mM NaCl, and 5 mM EDTA plus Complete protease inhibitors), passed repeatedly through a blunt needle, and clarified by centrifuging at 2000 x g for 1 minute. The PrP^C^ substrate was newly prepared for each independent experiment.

Prion-infected brain homogenates (strains RML, ME7, and mCWD) were of the same origin for all experiments and were used to seed clPMCA reactions containing mouse PrP^C^. Prion-infected brain homogenate (10% w/v) was added to PrP^C^-expressing RK13 cell lysate (1:10 PrP^Sc^: PrP^C^ by volume) and the lysate was subjected to repeated 5 seconds of sonication pulses (S4000, QSonica), with 10 minutes between each pulse, over 24 hours. Sonication power was maintained at 50–60% and samples were continuously rotated in a water bath at 37°C. Samples were then digested with 200 μg/ml PK for 30 minutes at 37°C and analyzed by western blot using the anti-PrP monoclonal antibody 3F4 [75]. PrP^C^ and PrP^Sc^ levels in unseeded lysate and samples, respectively, were measured by western blot and PrP^Sc^ signals compared to the no heparin control samples (considered 100%). PK-digested unseeded lysates were included in all experiments to exclude PrP^Sc^ contamination of the PMCA substrates and spontaneous assembly of mutant PrP^C^ protein. At least three independent experimental replicates were performed for each mutant and each prion strain used as seed. For the PCR assays with heparin (Scientific Protein Laboratories) and desulfated heparin (Tega), 1 μl of 225 μg/ml or 2,225 μg/ml sulfated heparin, or 1 μl of 225 μg/ml desulfated heparin (N-, 6-O-, or 2-O-desulfated) were added to the PMCA reactions. The level of PrP^Sc^ formed in the presence and absence of heparin was measured by western blot.

### h-FTAA staining and fluorescence life time imaging

Sections (10 μm) of OCT-embedded brain samples were cut onto positively charged silanized glass slides, dried for 1 hour, and fixed in 100% then 70% ethanol for 10 minutes each. After washing with deionized water, sections were equilibrated in PBS, pH 7.4, for 10 minutes. Heptamer-formyl thiophene acetic acid (h-FTAA; 1.5 mM in de-ionized water) was diluted in PBS to a final concentration of 1.5 μM and added to the sections. The sections were incubated with h-FTAA for 30 minutes at room temperature, washed with PBS, and mounted using Dako fluorescence mounting medium. The fluorescence decay of h-FTAA bound to PrP^Sc^ was collected using an inverted Zeiss (Axio Observer.Z1) LSM 780 microscope (Carl Zeiss MicroImaging GmbH) equipped with a modular FLIM system from Becker and Hickl. The emitted photons are routed through the direct coupling confocal port of the Zeiss LSM 780 scanning unit and detected by a Becker and Hickl HPM-100-40 hybrid detector. Data was recorded by a Becker and Hickl Simple-Tau 152 system (SPC-150 TCSPC FLIM module) with the instrument recording software SPCM version 9.42 in the FIFO image mode, 256 × 256 pixels, using 256 time channels (Becker and Hickl GmbH). For all acquisitions, a T80R20 main beam splitter was used, and the pinhole was set to 20.2 μm. A 490 nm laser line from a pulsed tunable In Tune laser (Carl Zeiss MicroImaging GmbH) with a repetition rate of 40 MHz was used for excitation. Data was subsequently analyzed in SPCImage version 3.9.4 (Becker and Hickl GmbH), fitting each of the acquired decay curves to a tri-exponential function and color-coded images, as well as distribution histograms, showing the intensity-weighted mean lifetimes generated with the same software. The procedure of staining and FLIM imaging of protein aggregates with h-FTAA has been described in detail [76].

### Purification of PrP^Sc^ for mass spectrometry and electron microscopy

To analyze HS bound to PrP^Sc^, PrP^Sc^ was first purified from mouse brains as previously described [46,77]. Briefly, 1 ml of 10% brain homogenate was mixed with an equal volume of 5% sarkosyl in TEN(D) buffer (50 mM Tris-HCl, 5 mM EDTA, 665 mM NaCl, 0.2 mM dithiothreitol, pH 8.0), containing Complete protease inhibitors (Roche). Samples were incubated on ice for 1 hour and centrifuged at 18,000 x g for 30 minutes at 4°C. All but 100 μl of supernatant was removed, and the pellet was resuspended in 100 μl of residual supernatant and diluted to 1 ml with 10% sarkosyl in TEN(D). Supernatants and pellets were incubated for 30 minutes on ice and then centrifuged at 18,000 x g for 30 minutes at 4°C. Supernatants were recovered while pellets were held on ice. Supernatants were added separately into ultracentrifuge tubes with 10% sarcosyl in TEN(D) buffer containing protease inhibitors and ultracentrifuged at 150,000 x g for 2.5 hours at 4°C. Supernatants were discarded while pellets were rinsed with 100 μl of 250 mM NaCl in TEN(D) buffer with 1% sulfobetaine (SB 3–14) and protease inhibitors and then combined and centrifuged at 200,000 x g for 2 hours at 20°C. The supernatant was discarded, and pellet was washed and then resuspended in 200 μl of ice cold TMS buffer (10 mM Tris-HCl, 5 mM MgCl_2_, 100 mM NaCl, pH 7.0) with protease inhibitors. Samples were incubated on ice overnight at 4°C. Samples were needle triturated and incubated with 25 units/ml nuclease (benzonase, Sigma-Aldrich) and 50 mM MgCl_2_ for 30 minutes at 37°C followed by a digestion with 1 mg/ml PK (final concentration) for 1 hour at 37°C. PK digestion was stopped by incubating samples with 2 mM PMSF on ice for 15 minutes. Samples were incubated with 2 mM EDTA for 15 minutes at 37°C, and then TMS buffer was added, prior to layering over a sucrose cushion (0.5 M sucrose, 10 mM Tris-HCl, pH 7.4, 100 mM NaCl, and 0.5% SB 3–14,) and centrifuging at 200,000 x g for 2 hours at 20°C. The pellet was rinsed and then resuspended in 0.5% SB 3–14 in PBS. Gel electrophoresis and silver staining as well as western blotting of sample and recPrP were performed to assess the quantity and purity of PrP^Sc^.

### Heparan sulfate purification and analysis by mass spectrometry

Heparan sulfate (HS) was extracted from whole brain homogenates and purified by anion exchange chromatography as previously described [46]. For depolymerization, HS chains were extensively digested with 1 milliunit each of heparin lyases I, II, and III (AMS Biotechnology). The disaccharides resulting from enzymatic depolymerization were tagged by reductive amination with [^12^C_6_] aniline and mixed with [^13^C_6_] aniline-tagged disaccharide standards. Samples were analyzed by liquid chromatography-mass spectrometry (LC-MS) using an LTQ Orbitrap Discovery electrospray ionization mass spectrometer (ThermoFisher Scientific). The disaccharides measured were: D0H0, D0A0, D0H6, D2H0, D0S0, D0A6, D2A0, D2H6, D0S6, D2S0, D2A6, and D2S6.

### Negative stain electron microscopy

400 mesh lacey carbon grids (Ted Pella) were glow discharged, placed on a 7 μl droplet of purified PrP^Sc^ sample, and incubated for 20 minutes in a humidified chamber. Grids were then blotted on filter paper, immersed briefly in Nano-W stain (Nanoprobes) and blotted again before incubating on a droplet of Nano-W for 1 minute. Grids were then blotted dry and imaged on a FEI Tecnai TF20 (200kV, FEG) with a 4k x 4k CMOS-based Tietz TemCam-F416 camera. Fibrils purified from terminally ill mCWD-infected *Ndst1*^*f/f*^*tga20*^*+/+*^*SynCre-* and *SynCre+* brains were distributed on the electron microscopy grids as single filaments as well as clusters of fibrils. Fibril lengths were assessed by three blinded investigators, and lengths recorded using Image J or iMOD software packages. Fibrils were only measured when both ends of a non-overlapping filament were readily visualized.

### PrP^C^ mutant generation and heparin sepharose chromatography

To identify the HS-binding domains, three clusters of lysine- and arginine- residues and one cluster of asparagine residues were exchanged for alanine within mouse *Prnp* in a pcDNA3.1 vector by site-directed mutagenesis (QuikChange site-directed mutagenesis kit; Agilent). PrP-deficient RK13 cells (ATCC) were transfected with 10 μg of plasmid DNA using Lipofectamine 3000 (Invitrogen). At 24 hours post-transfection, cells were washed twice in PBS and digested with 0.75 milliunits of phospholipase C from *Bacillus cereus* (Sigma Aldrich) in 1.5 ml of Opti-MEM media in PBS (1:2 dilution) (ThermoFisherScientific) for 1 hour at 37°C. The media was recovered and clarified by centrifugation at 2000 x g for 1 minute. Supernatants from the duplicate plates were pooled and saved for chromatography analysis.

For affinity chromatography, Heparin Sepharose 6 Fast Flow beads (Healthcare Life Sciences) were loaded into Bio-Spin chromatography columns (Bio-Rad) and packed with 2 ml of equilibration buffer (0.15 M NaCl in 25 mM HEPES, pH 7.4). Supernatants containing GPI-cleaved proteins, including WT and mutant PrP^C^, were applied onto the columns. The flow through was recovered, recirculated onto the column two times, and saved. The column was next washed with 2 ml of 0.15 M NaCl in 25 mM HEPES buffer (pH 7.4) and the unbound proteins were recovered in a clean tube. The bound PrP^C^ was step-eluted with 1 ml of elution buffer containing increasing concentrations of NaCl (300 mM– 2 M) in 25 mM HEPES. The unbound PrP^C^ in the 0.15 M wash and PrP^C^ in all eluates were analyzed for PrP^C^ level by immunoblot using POM19 antibody. At least three experimental replicates were performed for each PrP^C^ construct.

### RT-QuIC analysis of spinal cord lysates from mCWD-inoculated mice

The RT-QuIC reaction mix was composed of 10 mM phosphate buffer (pH 7.4), 130 mM NaCl, 0.1 mg/ml recombinant Syrian golden hamster prion protein (residues 90–231; rPrP^Sen^), 10 μM thioflavin T (ThT), 1 mM ethylenediaminetetraacetic acid tetrasodium salt (EDTA), and 0.002% SDS. Each well of a black 96-well plate with a clear bottom (Nunc) was loaded with aliquots of reaction mix (98 μl) and seeded with 2 μl of a 10^−2^ to 10^−4^ dilution of 10% mCWD spinal cord homogenate. The plate was sealed (plate sealer film, Nalgene Nunc International), incubated at 50°C in a BMG FLUOstar Omega plate reader and subjected to cycles of 1 min shaking (700 rpm double orbital) and 1 min rest with ThT fluorescence measurements (450 ± 10 nm excitation and 480 ± 10 nm emission; bottom read) taken every 45 minutes. Reactions were classified as RT-QuIC positive based on a threshold set at 10% of the maximum ThT fluorescence value on each plate.

### RecPrP conjugation to deferoxamine-maleimide

To label recPrP with zirconium-89, full length recombinant mouse PrP (23–230) generated in *E*.*coli* was first conjugated to deferoxamine-maleimide (Macrocyclics) to produce deferoxamine-conjugated PrP (DFO-recPrP). To ensure that the linker was not conjugated to the lysine-rich heparin binding domain, recPrP was bound to heparin sepharose beads to block the heparin binding sites. To block the binding sites, Heparin Sepharose 6 Fast Flow beads (1 ml) (Healthcare Life Sciences) were loaded into disposable Bio-Spin chromatography columns (Bio-Rad) and packed with 2 ml of equilibration buffer (0.15 M NaCl in 25 mM HEPES, pH 7.4). RecPrP (200 μg) was mixed with 1 ml of equilibration buffer and applied onto the columns. The columns were washed with 3 ml of equilibration buffer. The beads were then transferred to a 1.5 ml eppendorf polypropylene tube and incubated with 200 μl of deferoxamine-maleimide for 24 hours at room temperature with rotation. The bead slurry was transferred to a chromatography column and the recPrP conjugation was stopped after 24 hours by adding 600 μl PBS with 0.1 M glycine (pH 7). The beads were washed with 2 ml equilibration buffer, and the conjugated recPrP (DFO-recPrP) was eluted with 1 ml of elution buffer (0.7 M NaCl, 25 mM HEPES, pH 7.2). DFO-recPrP was concentrated using Zeba spin desalting columns.

### DFO-recPrP radiolabeling with zirconium-89

Zirconium-89 (Zr89) oxalate (Washington University) adjusted to pH 7.5 with 0.5 M HEPES/ Na_2_CO_3_ (2 M) solution was incubated with 30 μg deferoxamine-conjugated recPrP at room temperature for 60 minutes and diluted to approximately 100 μCi per injection following previously published procedures [78]. To measure the free Zr89 that had not been chelated, quality control was performed by instant thin layer chromatography (silica gel; 0.1 M citrate buffer pH 4.5); typical radiochemical purities (RF = 0.8) of 95–99% were achieved. Typical specific volume was 25 μCi/μl. To confirm that recPrP remained radiolabeled, four days after radiolabeling, Zr89-recPrP at different dilutions was loaded in 10% Bis-Tris gel (Invitrogen) and electrophoresed. As a marker, a small volume of Zr89 was placed on the protein ladder at 25 kDa, the expected size for recPrP, and the gel was scanned in a phosphor imager (Typhoon).

### Stereotaxic injection of conjugated recPrP

Mice (14–16 weeks old) (n = 3–4 mice/genotype/experiment) were weighed, anesthetized with isoflurane, and placed in a three-point stereotaxic apparatus (Stoelting). A 2 cm midline incision was made in the skin over the sagittal suture to expose bregma, and a burr hole was drilled in the left parietal bone (0.62 mm caudal, -1.75 mm lateral to bregma) using an Ideal Micro-drill. A 22-gauge needle (Hamilton) was inserted to a depth of 3.5 mm and 1 μl of Zr89-recPrP was injected at a rate of 75 nl/minute over 15 minutes using a Quintessential Stereotaxic Injector (Stoelting). The needle remained in the injection site for 10 minutes post-injection prior to removal from the brain to prevent backflow. Animals were removed from the stereotaxic device and placed on the PET scanner (G.E. Vista), and radioactivity measurements were collected as dynamic scans using list mode over 30 minutes. Scans were repeated 20 hours later. PET images were reconstructed using Vista DR (G.E. Healthcare) software, and the area and volume of radioactive signal, as well as the signal intensity, were assessed with an ImageJ based image processing software (FIJI).

### Statistics

Log-rank (Mantel-Cox) tests were performed to assess survival differences between groups. A Student’s t-test (two-tailed, unpaired) with Bonferroni’s post test was used to determine the statistical significance between the *Ndst1*^*f*/*f*^*SynCre*^*+/-*^ and *SynCre*^*-/-*^ and *Ndst1*^*f*/*f*^*GFAPCre*^*+/-*^ and *GFAPCre*^*-/-*^ mouse groups for the PrP^C^ level of expression, lesion profiles, activated microglia, PrP^Sc^ glycoprofiles, PrP^Sc^ conformation stability, and PrP^Sc^ fibril structure. One-way ANOVA with Tukey’s post test was performed to determine statistical significance in the levels of prion conversion by PMCA. Two-way ANOVA with Bonferroni’s post test was used to compare the composition of HS associated with different prion strains, the number of vascular versus parenchymal plaques, the heparin binding affinity of PrP^C^ mutants, and for PET scan experiments, the levels of recPrP in the spinal cord, and the area of high PET intensity signal (signal > 100 μCi). Unpaired two-tailed t-test with Bonferroni’s post-test was used to compare the average sulfate groups per disaccharide as well as the plaque length and the mCWD fibril length and solubility in *Ndst1*^*f*/*f*^*SynCre*^*+/-*^ and *SynCre*^*-/-*^ brain. The proportion of *Ndst1*^*f/f*^*SynCre*^*+/-*^ versus *SynCre*^*-/-*^ mice with prion seeding in the spinal cord was compared using non-parametric Fisher’s exact tests. For all analyses, *p* < 0.05 was considered significant.

## Supporting information

S1 Fig PrP^Sc^ selectively binds 6-O-sulfated HS in cerebellar samples from Gerstmann-Straüssler-Scheinker (GSS)-affected patients. Mass spectrometry analysis of HS bound to PrP^Sc^ and brain lysate from the same sample (the former published [46]). Quantification shows (A), unsulfated (NAc and NH_2_) and sulfated (NS, 2S, 6S) HS, (B), individual HS disaccharides, and (C), the average sulfation per disaccharide of HS. *N* = 3 cerebellar samples. Note: other disaccharides were not significantly different (S4 Table). *P< 0.05, **P< 0.01, ***P< 0.005 and ****P< 0.001, one-way ANOVA with Tukey’s post test (panels A and B), and unpaired two-tailed t-test with Bonferroni’s post test (panel C). NAc: N-acetylglucosamine (GlcNAc); NH_2_: glucosamine (GlcNH_2_); NS: N-sulfated glucosamine (GlcNS); 2S: 2-O-sulfated glucuronic or iduronic acids (2-O-S); 6S: 6-O-sulfated glucosamine (6-O-S).
(TIF)

S2 Fig PrP^C^ has two lysine-rich N-terminal heparin binding domains. (A) Mouse PrP amino acid sequence with alanine substitutions highlighted (Lys-, Arg-, and Asn- to Ala). The 3F4 epitope tag detected by anti-PrP antibody 3F4 (Leu- to Met and Val- to Met) is also shown. (B) Schematic representation of the heparin sepharose chromatography columns packed with heparin-coated beads. Heparin-bound mutant and WT PrP were step eluted using increasing concentrations NaCl. (C, D) Representative immunoblots and quantification of PrP recovered after each NaCl elution to assess the heparin binding affinity of mouse WT PrP compared to mutant PrP. While 0.3 M of NaCl is sufficient to elute most of the WT PrP bound to heparin (red arrow), Lys- and Arg- to Ala PrP mutants either run through the column without binding heparin (unbound) or are eluted with 0.15 M NaCl (C). *N* = 3–4 samples per PrP mutant. *P< 0.05, **P< 0.01 and ***P< 0.005, Two-way ANOVA with Bonferroni’s post test (panels C and D). Panel B created with BioRender.com.
(TIF)

S3 Fig Uninfected *Ndst1*^*f/f*^*tga20*^*+/+*^*SynCre+* mice express less sulfated HS and show lower microglial reactivity in the brain. (A) Schematic representation of the breeding strategy to generate *Ndst1*^*f/f*^*tga20*^*+/+*^*SynCre*^*+/-*^ mice and littermate controls. (B) HS in whole brain lysates from uninfected *SynCre-* and *SynCre+* mice. Quantification of unsulfated (NAc and NH_2_) and sulfated (NS, 2S, 6S) HS and (C) average sulfation per HS disaccharide (*n* = 5 *SynCre-* and 6 *SynCre+* brains). (D) Representative images of hematoxylin-eosin (HE)-stained, astrocyte (GFAP)-immunolabelled, and (E) microglia (Iba1)-immunolabelled brain sections (hippocampus). Scale bar = 100 μm. (F) Quantification of Iba1-immunolabelled over total area. *N* = 6 samples per group. CC: corpus callosum, CT: cortex, TH: thalamus, HP: hippocampus and CB: cerebellum. (G) PrP immunoblots and (H) quantification of PrP^C^ levels in *SynCre-* and *SynCre+* mice. *N* = 3 samples per group. *P< 0.05, **P< 0.01 and ****P< 0.001, two-way ANOVA with Bonferroni’s post test (panels B and F), and unpaired two-tailed t-test with Bonferroni’s post test (panels C and H). NAc: N-acetylglucosamine (GlcNAc); NH_2_: glucosamine (GlcNH_2_); NS: N-sulfated glucosamine (GlcNS); 2S: 2-O-sulfated glucuronic or iduronic acids (2-O-S); 6S: 6-O-sulfated glucosamine (6-O-S). Panel A created with BioRender.com.
(TIF)

S4 Fig *Ndst1*^*f/f*^*SynCre-* and *SynCre+* mice infected with subfibrillar prions: survival times, brain lesions, and biochemical properties. (A) Survival curves of *SynCre-* and *SynCre+* mice intracerebrally inoculated with RML or ME7 prions (RML: *n* = 7 *SynCre-* and 8 *SynCre+* mice; ME7: *n* = 16 *SynCre-* and 17 *SynCre+* mice). (B) Brain sections (hippocampus) immunolabelled for PrP^Sc^ or GFAP or stained with hematoxylin and eosin (HE) show prion aggregate distribution and morphology, spongiform degeneration, and astrogliosis in *SynCre-* and *SynCre+* brains. Scale bar = 50 μm. (C) Lesion profiles of RML- and ME7-infected *SynCre-* and *SynCre+* mice (1-dorsal medulla, 2-cerebellum, 3-hypothalamus, 4-medial thalamus, 5-hippocampus, 6-septum, 7-cerebral cortex, 8- cerebral peduncle and 9- cerebellar peduncle) (RML: *n* = 5 mice per genotype; ME7: *n* = 6 *SynCre-* and 7 *SynCre+* mice). (D) PrP^Sc^ immunoblots (post-PK digest) show electrophoretic mobility and glycoprofile of PK-digested PrP^Sc^. *N* = 6 per genotype. (E) Immunolabelling for microglia (Iba1) (representative images of hippocampus) and (F) quantification of the Iba1-stained area in CC, CT, HP, TH. Scale bar = 50 μm. (G) Representative immunoblots for PrP^Sc^ (post PK digest) from 30 μg brain, and (H) quantification of PrP^Sc^ levels in the whole brain lysate at terminal disease. *P< 0.05, log-rank (Mantel-Cox) test (panel A), unpaired two-tailed t-test with Bonferroni’s post test (panels D and H), and two-way ANOVA with Bonferroni’s post test (panel F). CC: corpus callosum, CT: cerebral cortex, TH: thalamus and HP: hippocampus; PK: proteinase K.
(TIF)

S5 Fig Lowering neuronal HS sulfation reduces parenchymal prion plaques in mCWD-infected mice. Quantification of (A) parenchymal and (B) vascular plaques in brain sections immunolabelled for PrP and endothelial cells (CD31) (representative sections shown in Fig 2G). *N* = 6 *SynCre-* and 9 *SynCre+* mice. (C) Representative images of hippocampus from *SynCre-* and *SynCre+* mice show immunolabeling for astrocytes (GFAP) and microglia (Iba1). (D) Quantification of the GFAP- and Iba1-immunolabelled area in C. Scale bar = 50 μm. *N =* 7 *SynCre-* mice and 8 *SynCre+* mice. (E) Dual immunostaining of mCWD-infected brain sections for PrP and HS shows mCWD plaques label strongly for HS [corpus callosum (*SynCre-*) and thalamus (*SynCre+*)]. Pre-treating brain sections with heparinases abolished HS labelling. Scale bar = 50 μm. (F) Electron microscopy images of mCWD plaques in corpus callosum (*SynCre-*) and velum interpositum (*SynCre+*). The dense core is highlighted with an asterisk. Scale bar = 5 μm. ***P< 0.005, two-way ANOVA with Bonferroni’s post test (panels A, B and D). CC: corpus callosum, HP: hippocampus, VI: velum interpositum, TH: thalamus, HT: hypothalamus, BG: basal ganglia, CT: cerebral cortex, and CB: cerebellum.
(TIF)

S6 Fig Reducing neuronal HS sulfation does not alter mCWD plaque-bound h-FTAA lifetime decay, nor the PrP^Sc^ glycoprofile or stability in chaotropes. (A) Representative images and curves of the h-FTAA fluorescence life-time decay of mCWD prion plaques in *Ndst1*^*f/f*^*tga20*^*+/+*^*SynCre-* and *SynCre+* brain sections. *N* = 5 *SynCre-* mice and 3 *SynCre+* mice. (B) PrP^Sc^ (mCWD) electrophoretic mobility and (C) glycoprofile in *SynCre-* and *SynCre+* mice. *N* = 6 *SynCre-* and 7 *SynCre+* mice. (D) Representative example of PrP^Sc^ stability as measured by GdnHCl denaturation in mCWD-infected *SynCre-* and *SynCre+* mice. *N* = 4 brain samples per genotype. (E) [GdnHCl]_1/2_ for PrP^Sc^ in *SynCre-* and *SynCre+* brain. ps = picoseconds (panel A). Unpaired two-tailed t-test with Bonferroni’s post test (panels A, C, and E).
(TIF)

S7 Fig Zr89-recPrP signal quantification from PET scans of live *Ndst1*^*f/f*^*tga20*^*+/+*^*SynCre-* and *SynCre+* mice. (A) Phosphor-imaged gel following electrophoresis of Zr89-recPrP (1:3 dilution series) four days after radiolabeling shows that recPrP was not degraded and remained radiolabeled. Zr89 was placed on the precision ladder at the expected size for recPrP (green arrow, 25 kDa). (B, C) Quantified signal from sagittal brain sections immediately after Zr89-recPrP injection (D0) show that (B) the total PET signal area and (C) the high signal intensity (>100 μCi) area for two experiments (Exp) are similar in *SynCre-* and *SynCre+* mice. (D) Total PET signal area at day 1 (D1; 20 hours later) is 60 to 80% increased in *SynCre+* brain versus only increased by 5% in *SynCre-* brain in two experiments. *P< 0.05 and **P< 0.01, two-way ANOVA with Bonferroni’s post test (panels B-D). *N* = 3 mice per genotype for each experiment.
(TIF)

S8 Fig Reduced astrocytic HS sulfation does not alter prion disease progression or disease phenotype. Quantification of (A) unsulfated (NAc and NH_2_) and sulfated (NS, 2S, 6S) HS, (B) individual HS disaccharides, and (C) average sulfation per HS disaccharide in whole brain lysates from approximately 420 day old *Ndst1*^*f/f*^*tga20*^*+/+*^*GFAPCre-* and *Cre+* brain samples (*n* = 5 *GFAPCre-* and 6 *Cre+* brain samples). Other disaccharides shown in S8 Table. (D) Immunoblotting for PrP^C^ levels in *GFAPCre+* and *Cre-* brain. *N =* 3 brains per genotype. (E) Quantification of GFAP- and (F) Iba1-immunolabelled area (*n* = 4–7 mice per group per stain). (G) Survival curves for ME7- and RML-inoculated *Ndst1*^*f/f*^*GFAPCre+* and *Cre-* mice in ME7-inoculated *Ndst1*^*f/f*^*GFAPCre+* (ME7: *n =* 7 *GFAPCre-* mice and 11 *Cre+* mice; RML: *n* = 11 *GFAPCre-* and 10 *Cre+* mice). (H) Brain sections (hippocampus) immunolabelled for PrP or GFAP, or stained with hematoxylin and eosin (HE), and (I) lesion profiles for ME7- and RML- infected *GFAPCre+* brain (ME7: *n* = 5 *GFAPCre-* and 4 *Cre+* mice; RML: *n* = 5 *GFAPCre-* and 6 *Cre+* mice). (J) Electrophoretic mobility and glycoprofile of PrP^Sc^ from ME7- and RML-infected *Ndst1*^*f/*^*GFAPCre-* brain (ME7: n = 6 *GFAPCre-* and 8 *Cre+* brains; RML: n = 8 brains per genotype). (K) mCWD-infected *Ndst1*^*f/f*^*tga20*^*+/+*^*GFAPCre+* and *Cre-* mice survival times (*n* = 12 *GFAPCre-* and 16 *Cre+* mice). (L) Spongiform change [HE stains (arrowheads depict plaques), PrP^Sc^, and astrocyte immunolabelling (GFAP)], (M) plaque distribution (*n* = 9 *GFAPCre-* and 11 *Cre+* brains), and (N) microglial response (*n* = 8 *GFAPCre-* and 11 *GFAPCre+* brains), the latter quantified in (O) in *GFAPCre+* and *Cre-* mice. (P) The electrophoretic mobility, glycoprofile (*n* = 6 *GFAPCre-* and 7 *Cre+* brains) and (Q) PrP^Sc^-bound h-FTAA fluorescence life-time decay in brain (*n* = 5 *GFAPCre-* and 6 *Cre+*). Two-way ANOVA with Bonferroni’s post test (panels A-C, E, F, M and O), and unpaired two-tailed t-test with Bonferroni’s post test (panels D, J, P and Q), and Log-rank (Mantel-Cox) test (panel G and K). NAc: N-acetylglucosamine (GlcNAc); NH_2_: glucosamine (GlcNH_2_); NS: N-sulfated glucosamine (GlcNS); 2S: 2-O-sulfated glucuronic or iduronic acids (2-O-S); 6S: 6-O-sulfated glucosamine (6-O-S).
(TIF)

S1 Table Disaccharide composition of heparan sulfate molecules bound to PrP^Sc^ versus brain lysate of ME7-infected mice. (PDF)

S2 Table Disaccharide composition of heparan sulfate molecules bound to PrP^Sc^ versus brain lysate of mCWD-infected mice. (PDF)

S3 Table Disaccharide composition of heparan sulfate molecules bound to PrP^Sc^ versus brain lysate of sCJD patients. (PDF)

S4 Table Disaccharide composition of heparan sulfate molecules bound to PrP^Sc^ versus brain lysate of GSS patients. (PDF)

S5 Table Disaccharide composition of heparan sulfate molecules in uninfected *Ndst1*^*f/f*^*tga20*^*+/+*^*SynCre*- and *SynCre*+ brain. (PDF)

S6 Table Quantification of mCWD parenchymal and vascular plaques in mCWD-infected *Ndst1*^*f/f*^*tga20*^*+/+*^*SynCre*- and *SynCre*+ brain. (PDF)

S7 Table mCWD seeding activity in the spinal cord of *Ndst1*^*f/f*^*tga20*^*+/+*^*SynCre*- and *SynCre*+ mice using real-time quaking-induced conversion. (PDF)

S8 Table Disaccharide composition of heparan sulfate molecules in *Ndst1*^*f/f*^*tga20*^*+/+*^*GFAPCre*- and *GFAPCre*+ brain. (PDF)

## References

[1] Henderson MX, Cornblath EJ, Darwich A, Zhang B, Brown H, Gathagan RJ, et al. Spread of α-synuclein pathology through the brain connectome is modulated by selective vulnerability and predicted by network analysis. Nat Neurosci. 2019;22(8):1248–57. doi: 10.1038/s41593-019-0457-5 PMCID: PMC6662627 31346295 PMC6662627

[2] Jucker M, Walker LC. Self-propagation of pathogenic protein aggregates in neurodegenerative diseases. Nature. 2013;501(7465):45–51. doi: 10.1038/nature12481 24005412 PMC3963807

[3] Thal DR, Rüb U, Orantes M, Braak H. Phases of A beta-deposition in the human brain and its relevance for the development of AD. Neurology. 2002;58(12):1791–800. doi: 10.1212/wnl.58.12.1791 12084879

[4] Raj A, LoCastro E, Kuceyeski A, Tosun D, Relkin N, Weiner M. Network Diffusion Model of Progression Predicts Longitudinal Patterns of Atrophy and Metabolism in Alzheimer’s Disease. Cell reports. 2015;10(3):359–69. doi: 10.1016/j.celrep.2014.12.034 PMCID: PMC5747552 25600871 PMC5747552

[5] Aguzzi A, Rajendran L. The transcellular spread of cytosolic amyloids, prions, and prionoids. Neuron. 2009;64(6):783–90. doi: 10.1016/j.neuron.2009.12.016 20064386

[6] Beekes M, McBride PA, Baldauf E. Cerebral targeting indicates vagal spread of infection in hamsters fed with scrapie. J Gen Virol. 1998;79 Part 3:601–7. doi: 10.1099/0022-1317-79-3-601 9519840

[7] Iliff JJ, Wang M, Zeppenfeld DM, Venkataraman A, Plog BA, Liao Y, et al. Cerebral arterial pulsation drives paravascular CSF-interstitial fluid exchange in the murine brain. J Neurosci. 2013;33(46):18190–9. doi: 10.1523/JNEUROSCI.1592-13.2013 24227727 PMC3866416

[8] Carare RO, Bernardes-Silva M, Newman TA, Page AM, Nicoll JA, Perry VH, et al. Solutes, but not cells, drain from the brain parenchyma along basement membranes of capillaries and arteries: significance for cerebral amyloid angiopathy and neuroimmunology. Neuropathol Appl Neurobiol. 2008;34(2):131–44. doi: 10.1111/j.1365-2990.2007.00926.x 18208483

[9] Iliff JJ, Chen MJ, Plog BA, Zeppenfeld DM, Soltero M, Yang L, et al. Impairment of glymphatic pathway function promotes tau pathology after traumatic brain injury. J Neurosci. 2014;34(49):16180–93. doi: 10.1523/JNEUROSCI.3020-14.2014 25471560 PMC4252540

[10] Iliff JJ, Lee H, Yu M, Feng T, Logan J, Nedergaard M, et al. Brain-wide pathway for waste clearance captured by contrast-enhanced MRI. J Clin Invest. 2013;123(3):1299–309. doi: 10.1172/JCI67677 23434588 PMC3582150

[11] Iliff JJ, Wang M, Liao Y, Plogg BA, Peng W, Gundersen GA, et al. A paravascular pathway facilitates CSF flow through the brain parenchyma and the clearance of interstitial solutes, including amyloid beta. Sci Transl Med. 2012;4(147):147ra11. doi: 10.1126/scitranslmed.3003748 PMCID: PMC3551275 22896675 PMC3551275

[12] Hawkes CA, Härtig W, Kacza J, Schliebs R, Weller RO, Nicoll JA, et al. Perivascular drainage of solutes is impaired in the ageing mouse brain and in the presence of cerebral amyloid angiopathy. Acta Neuropathol. 2011;121(4):431–43. doi: 10.1007/s00401-011-0801-7 21259015

[13] Kang JE, Lim MM, Bateman RJ, Lee JJ, Smyth LP, Cirrito JR, et al. Amyloid-beta dynamics are regulated by orexin and the sleep-wake cycle. Science. 2009;326(5955):1005–7. doi: 10.1126/science.1180962 19779148 PMC2789838

[14] Xie L, Kang H, Xu Q, Chen MJ, Liao Y, Thiyagarajan M, et al. Sleep drives metabolite clearance from the adult brain. Science. 2013;342(6156):373–7. doi: 10.1126/science.1241224 24136970 PMC3880190

[15] Sun Y, Xu S, Jiang M, Liu X, Yang L, Bai Z, et al. Role of the Extracellular Matrix in Alzheimer’s Disease. Front Aging Neurosci. 2021;13:707466. doi: 10.3389/fnagi.2021.707466 34512308 PMC8430252

[16] Kress BT, Iliff JJ, Xia M, Wang M, Wei HS, Zeppenfeld D, et al. Impairment of paravascular clearance pathways in the aging brain. Ann Neurol. 2014;76(6):845–61. doi: 10.1002/ana.24271 25204284 PMC4245362

[17] Nicholson C, Hrabětová S. Brain Extracellular Space: The Final Frontier of Neuroscience. Biophys J. 2017;113(10):2133–42. doi: 10.1016/j.bpj.2017.06.052 PMCID: PMC5700249 28755756 PMC5700249

[18] Prusiner SB. Novel proteinaceous infectious particles cause scrapie. Science. 1982;216(4542):136–44. doi: 10.1126/science.6801762 6801762

[19] Spraker TR, Zink RR, Cummings BA, Sigurdson CJ, Miller MW, O’Rourke KI. Distribution of protease-resistant prion protein and spongiform encephalopathy in free-ranging mule deer (Odocoileus hemionus) with chronic wasting disease. Vet Pathol. 2002;39(5):546–56. doi: 10.1354/vp.39-5-546 12243464

[20] Bruce ME. TSE strain variation. Br Med Bull. 2003;66:99–108. doi: 10.1093/bmb/66.1.99 14522852

[21] Gambetti P, Kong Q, Zou W, Parchi P, Chen SG. Sporadic and familial CJD: classification and characterisation. Br Med Bull. 2003;66:213–39. doi: 10.1093/bmb/66.1.213 14522861

[22] Su JH, Cummings BJ, Cotman CW. Localization of heparan sulfate glycosaminoglycan and proteoglycan core protein in aged brain and Alzheimer’s disease. Neuroscience. 1992;51(4):801–13. doi: 10.1016/0306-4522(92)90521-3 1488123

[23] Snow AD, Mar H, Nochlin D, Kimata K, Kato M, Suzuki S, et al. The presence of heparan sulfate proteoglycans in the neuritic plaques and congophilic angiopathy in Alzheimer’s disease. Am J Pathol. 1988;133(3):456–63. 2974240 PMC1880818

[24] Snow AD, Kisilevsky R, Willmer J, Prusiner SB, DeArmond SJ. Sulfated glycosaminoglycans in amyloid plaques of prion diseases. Acta Neuropathol Berl. 1989;77(4):337–42. doi: 10.1007/BF00687367 2523631

[25] McBride PA, Wilson MI, Eikelenboom P, Tunstall A, Bruce ME. Heparan sulfate proteoglycan is associated with amyloid plaques and neuroanatomically targeted PrP pathology throughout the incubation period of scrapie-infected mice. Exp Neurol. 1998;149(2):447–54. doi: 10.1006/exnr.1997.6740 9500966

[26] Sarrazin S, Lamanna WC, Esko JD. Heparan sulfate proteoglycans. Cold Spring Harb Perspect Biol. 2011;3(7). doi: 10.1101/cshperspect.a004952 PMCID: PMC3119907 21690215 PMC3119907

[27] Xu D, Esko JD. Demystifying heparan sulfate-protein interactions. Annu Rev Biochem. 2014;83:129–57. doi: 10.1146/annurev-biochem-060713-035314 24606135 PMC7851832

[28] Horonchik L, Tzaban S, Ben-Zaken O, Yedidia Y, Rouvinski A, Papy-Garcia D, et al. Heparan sulfate is a cellular receptor for purified infectious prions. J Biol Chem. 2005;280(17):17062–7. doi: 10.1074/jbc.M500122200 15668247

[29] Kanekiyo T, Zhang J, Liu Q, Liu CC, Zhang L, Bu G. Heparan sulphate proteoglycan and the low-density lipoprotein receptor-related protein 1 constitute major pathways for neuronal amyloid-beta uptake. J Neurosci. 2011;31(5):1644–51. doi: 10.1523/JNEUROSCI.5491-10.2011 21289173 PMC3272839

[30] Hudak A, Kusz E, Domonkos I, Josvay K, Kodamullil AT, Szilak L, et al. Contribution of syndecans to cellular uptake and fibrillation of alpha-synuclein and tau. Sci Rep. 2019;9(1):16543. doi: 10.1038/s41598-019-53038-z PMCID: PMC6851098 31719623 PMC6851098

[31] Letoha T, Hudak A, Kusz E, Pettko-Szandtner A, Domonkos I, Josvay K, et al. Contribution of syndecans to cellular internalization and fibrillation of amyloid-beta(1–42). Sci Rep. 2019;9(1):1393. doi: 10.1038/s41598-018-37476-9 PMCID: PMC6362000 30718543 PMC6362000

[32] Holmes BB, DeVos SL, Kfoury N, Li M, Jacks R, Yanamandra K, et al. Heparan sulfate proteoglycans mediate internalization and propagation of specific proteopathic seeds. Proc Natl Acad Sci U S A. 2013;110(33):E3138–47. doi: 10.1073/pnas.1301440110 23898162 PMC3746848

[33] Farquhar CF, Dickinson AG. Prolongation of scrapie incubation period by an injection of dextran sulphate 500 within the month before or after infection. J Gen Virol. 1986;67(Pt 3):463–73. doi: 10.1099/0022-1317-67-3-463 2419489

[34] Ehlers B, Diringer H. Dextran sulphate 500 delays and prevents mouse scrapie by impairment of agent replication in spleen. J Gen Virol. 1984;65(Pt 8):1325–30. doi: 10.1099/0022-1317-65-8-1325 6205119

[35] Ladogana A, Casaccia P, Ingrosso L, Cibati M, Salvatore M, Xi YG, et al. Sulphate polyanions prolong the incubation period of scrapie-infected hamsters. J Gen Virol. 1992;73(Pt 3):661–5. doi: 10.1099/0022-1317-73-3-661 1372039

[36] Caughey B, Raymond GJ. Sulfated polyanion inhibition of scrapie-associated PrP accumulation in cultured cells. J Virol. 1993;67(2):643–50. doi: 10.1128/JVI.67.2.643-650.1993 7678300 PMC237415

[37] Caughey B, Brown K, Raymond GJ, Katzenstein GE, Thresher W. Binding of the protease-sensitive form of PrP (prion protein) to sulfated glycosaminoglycan and congo red [corrected] [published erratum appears in J Virol 1994 Jun;68(6):4107]. J Virol. 1994;68(4):2135–41. doi: 10.1128/JVI.68.4.2135-2141.1994 PMCID: PMC236688 7511169 PMC236688

[38] Wong C, Xiong LW, Horiuchi M, Raymond L, Wehrly K, Chesebro B, et al. Sulfated glycans and elevated temperature stimulate PrP(Sc)-dependent cell-free formation of protease-resistant prion protein. EMBO J. 2001;20(3):377–86. doi: 10.1093/emboj/20.3.377 11157745 PMC133469

[39] Adjou KT, Simoneau S, Sales N, Lamoury F, Dormont D, Papy-Garcia D, et al. A novel generation of heparan sulfate mimetics for the treatment of prion diseases. J Gen Virol. 2003;84(Pt 9):2595–603. doi: 10.1099/vir.0.19073-0 12917481

[40] Doh-ura K, Ishikawa K, Murakami-Kubo I, Sasaki K, Mohri S, Race R, et al. Treatment of transmissible spongiform encephalopathy by intraventricular drug infusion in animal models. J Virol. 2004;78(10):4999–5006. doi: 10.1128/jvi.78.10.4999-5006.2004 15113880 PMC400350

[41] Todd NV, Morrow J, Doh-ura K, Dealler S, O’Hare S, Farling P, et al. Cerebroventricular infusion of pentosan polysulphate in human variant Creutzfeldt-Jakob disease. J Infect. 2005;50(5):394–6. doi: 10.1016/j.jinf.2004.07.015 15907546

[42] Parry A, Baker I, Stacey R, Wimalaratna S. Long term survival in a patient with variant Creutzfeldt-Jakob disease treated with intraventricular pentosan polysulphate. J Neurol Neurosurg Psychiatry. 2007;78(7):733–4. doi: 10.1136/jnnp.2006.104505 17314188 PMC2117700

[43] Larramendy-Gozalo C, Barret A, Daudigeos E, Mathieu E, Antonangeli L, Riffet C, et al. Comparison of CR36, a new heparan mimetic, and pentosan polysulfate in the treatment of prion diseases. J Gen Virol. 2007;88(Pt 3):1062–7. doi: 10.1099/vir.0.82286-0 17325382

[44] Newman PK, Todd NV, Scoones D, Mead S, Knight RS, Will RG, et al. Postmortem findings in a case of variant Creutzfeldt-Jakob disease treated with intraventricular pentosan polysulfate. J Neurol Neurosurg Psychiatry. 2014;85(8):921–4. doi: 10.1136/jnnp-2013-305590 24554103 PMC4112497

[45] Kovalchuk Ben-Zaken O, Nissan I, Tzaban S, Taraboulos A, Zcharia E, Matzger S, et al. Transgenic over-expression of mammalian heparanase delays prion disease onset and progression. Biochem Biophys Res Commun. 2015;464(3):698–704. doi: 10.1016/j.bbrc.2015.06.170 26168721 PMC4532585

[46] Aguilar-Calvo P, Sevillano AM, Bapat J, Soldau K, Sandoval DR, Altmeppen HC, et al. Shortening heparan sulfate chains prolongs survival and reduces parenchymal plaques in prion disease caused by mobile, ADAM10-cleaved prions. Acta Neuropathol. 2019. doi: 10.1007/s00401-019-02085-x 31673874 PMC7036335

[47] Castillo GM, Lukito W, Wight TN, Snow AD. The sulfate moieties of glycosaminoglycans are critical for the enhancement of beta-amyloid protein fibril formation. J Neurochem. 1999;72(4):1681–7. doi: 10.1046/j.1471-4159.1999.721681.x 10098877

[48] Cotman SL, Halfter W, Cole GJ. Agrin binds to beta-amyloid (Abeta), accelerates abeta fibril formation, and is localized to Abeta deposits in Alzheimer’s disease brain. Mol Cell Neurosci. 2000;15(2):183–98. doi: 10.1006/mcne.1999.0816 10673326

[49] Cohlberg JA, Li J, Uversky VN, Fink AL. Heparin and other glycosaminoglycans stimulate the formation of amyloid fibrils from alpha-synuclein in vitro. Biochemistry. 2002;41(5):1502–11. doi: 10.1021/bi011711s 11814343

[50] Bazar E, Jelinek R. Divergent heparin-induced fibrillation pathways of a prion amyloidogenic determinant. ChemBioChem. 2010;11(14):1997–2002. doi: 10.1002/cbic.201000207 20799315

[51] Zhu HL, Fernandez C, Fan JB, Shewmaker F, Chen J, Minton AP, et al. Quantitative characterization of heparin binding to Tau protein: implication for inducer-mediated Tau filament formation. J Biol Chem. 2010;285(6):3592–9. doi: 10.1074/jbc.M109.035691 19959468 PMC2824206

[52] Ellett LJ, Coleman BM, Shambrook MC, Johanssen VA, Collins SJ, Masters CL, et al. Glycosaminoglycan sulfation determines the biochemical properties of prion protein aggregates. Glycobiology. 2015;25(7):745–55. doi: 10.1093/glycob/cwv014 25701659

[53] Saborio GP, Permanne B, Soto C. Sensitive detection of pathological prion protein by cyclic amplification of protein misfolding. Nature. 2001;411(6839):810–3. doi: 10.1038/35081095 11459061

[54] Wadia JS, Schaller M, Williamson RA, Dowdy SF. Pathologic prion protein infects cells by lipid-raft dependent macropinocytosis. PLoS One. 2008;3(10):e3314. doi: 10.1371/journal.pone.0003314 19390657 PMC2671965

[55] Sigurdson CJ, Manco G, Schwarz P, Liberski P, Hoover EA, Hornemann S, et al. Strain fidelity of chronic wasting disease upon murine adaptation. J Virol. 2006;80(24):12303–11. doi: 10.1128/JVI.01120-06 17020952 PMC1676299

[56] Fischer M, Rulicke T, Raeber A, Sailer A, Moser M, Oesch B, et al. Prion protein (PrP) with amino-proximal deletions restoring susceptibility of PrP knockout mice to scrapie. EMBO J. 1996;15(6):1255–64. 8635458 PMC450028

[57] Allen NJ, Bennett ML, Foo LC, Wang GX, Chakraborty C, Smith SJ, et al. Astrocyte glypicans 4 and 6 promote formation of excitatory synapses via GluA1 AMPA receptors. Nature. 2012;486(7403):410–4. doi: 10.1038/nature11059 22722203 PMC3383085

[58] Condomitti G, de Wit J. Heparan Sulfate Proteoglycans as Emerging Players in Synaptic Specificity. Front Mol Neurosci. 2018;11:14. doi: 10.3389/fnmol.2018.00014 29434536 PMC5790772

[59] O’Callaghan P, Li JP, Lannfelt L, Lindahl U, Zhang X. Microglial Heparan Sulfate Proteoglycans Facilitate the Cluster-of-Differentiation 14 (CD14)/Toll-like Receptor 4 (TLR4)-Dependent Inflammatory Response. J Biol Chem. 2015;290(24):14904–14. doi: 10.1074/jbc.M114.634337 25869127 PMC4463438

[60] Snow AD, Wight TN, Nochlin D, Koike Y, Kimata K, DeArmond SJ, et al. Immunolocalization of heparan sulfate proteoglycans to the prion protein amyloid plaques of Gerstmann-Straussler syndrome, Creutzfeldt-Jakob disease and scrapie. Lab Invest. 1990;63(5):601–11. 1977959

[61] Liu CC, Zhao N, Yamaguchi Y, Cirrito JR, Kanekiyo T, Holtzman DM, et al. Neuronal heparan sulfates promote amyloid pathology by modulating brain amyloid-beta clearance and aggregation in Alzheimer’s disease. Sci Transl Med. 2016;8(332):332ra44. doi: 10.1126/scitranslmed.aad3650 PMCID: PMC5512541 27030596 PMC5512541

[62] Deleault NR, Harris BT, Rees JR, Supattapone S. Formation of native prions from minimal components in vitro. Proc Natl Acad Sci U S A. 2007;104(23):9741–6. doi: 10.1073/pnas.0702662104 17535913 PMC1887554

[63] Deleault NR, Piro JR, Walsh DJ, Wang F, Ma J, Geoghegan JC, et al. Isolation of phosphatidylethanolamine as a solitary cofactor for prion formation in the absence of nucleic acids. Proc Natl Acad Sci U S A. 2012;109(22):8546–51. doi: 10.1073/pnas.1204498109 22586108 PMC3365173

[64] Wang F, Wang X, Yuan CG, Ma J. Generating a prion with bacterially expressed recombinant prion protein. Science. 2010;327(5969):1132–5. doi: 10.1126/science.1183748 20110469 PMC2893558

[65] Fichou Y, Oberholtzer ZR, Ngo H, Cheng CY, Keller TJ, Eschmann NA, et al. Tau-Cofactor Complexes as Building Blocks of Tau Fibrils. Front Neurosci. 2019;13:1339. doi: 10.3389/fnins.2019.01339 31920504 PMC6923735

[66] Ihse E, Yamakado H, van Wijk XM, Lawrence R, Esko JD, Masliah E. Cellular internalization of alpha-synuclein aggregates by cell surface heparan sulfate depends on aggregate conformation and cell type. Sci Rep. 2017;7(1):9008. doi: 10.1038/s41598-017-08720-5 28827536 PMC5566500

[67] Kraus A, Hoyt F, Schwartz CL, Hansen B, Artikis E, Hughson AG, et al. High-resolution structure and strain comparison of infectious mammalian prions. Mol Cell. 2021;81(21):4540–51.e6. doi: 10.1016/j.molcel.2021.08.011 34433091

[68] Kamimura K, Maeda N. Glypicans and Heparan Sulfate in Synaptic Development, Neural Plasticity, and Neurological Disorders. Frontiers in neural circuits. 2021;15:595596. doi: 10.3389/fncir.2021.595596 33679334 PMC7928303

[69] Mallucci G, Dickinson A, Linehan J, Klohn PC, Brandner S, Collinge J. Depleting neuronal PrP in prion infection prevents disease and reverses spongiosis. Science. 2003;302(5646):871–4. doi: 10.1126/science.1090187 14593181

[70] Giulian D, Haverkamp LJ, Yu J, Karshin W, Tom D, Li J, et al. The HHQK domain of beta-amyloid provides a structural basis for the immunopathology of Alzheimer’s disease. J Biol Chem. 1998;273(45):29719–26. doi: 10.1074/jbc.273.45.29719 9792685

[71] Lindahl B, Westling C, Giménez-Gallego G, Lindahl U, Salmivirta M. Common binding sites for beta-amyloid fibrils and fibroblast growth factor-2 in heparan sulfate from human cerebral cortex. J Biol Chem. 1999;274(43):30631–5. doi: 10.1074/jbc.274.43.30631 10521448

[72] Grobe K, Inatani M, Pallerla SR, Castagnola J, Yamaguchi Y, Esko JD. Cerebral hypoplasia and craniofacial defects in mice lacking heparan sulfate Ndst1 gene function. Development. 2005;132(16):3777–86. doi: 10.1242/dev.01935 16020517 PMC7851831

[73] Wadsworth JDF, Joiner, S., Hill, A.F., Campbell, T.A., Desbruslais, M., Luthert, P.J., Collinge, J. Tissue distribution of protease resistant prion protein in variant CJD using a highly sensitive immuno-blotting assay. Lancet. 2001;358:171–80. doi: 10.1016/s0140-6736(01)05403-4 11476832

[74] Polymenidou M, Moos R, Scott M, Sigurdson C, Shi YZ, Yajima B, et al. The POM monoclonals: a comprehensive set of antibodies to non-overlapping prion protein epitopes. PLoS One. 2008;3(12):e3872. doi: 10.1371/journal.pone.0003872 19060956 PMC2592702

[75] Kascsak RJ, Rubenstein R, Merz PA, Tonna DeMasi M, Fersko R, Carp RI, et al. Mouse polyclonal and monoclonal antibody to scrapie-associated fibril proteins. J Virol. 1987;61(12):3688–93. doi: 10.1128/JVI.61.12.3688-3693.1987 2446004 PMC255980

[76] Nyström S, Bäck, M., Nilsson, K.P., Hammarström, P. Imaging Amyloid Tissues Stained with Luminescent Conjugated Oligothiophenes by Hyperspectral Confocal Microscopy and Fluorescence Lifetime Imaging. Journal of Visualized Experiments. 2017;128. doi: 10.3791/56279 PMCID: PMC5755170 29155738 PMC5755170

[77] Raymond GJ, Chabry J. Methods and Tools in Biosciences and Medicine. In: Lehmann S, Grassi J, editors. Techniques in Prion Research: Birkhäuser, Basel; 2004. p. 16–26.

[78] Bailly C, Gouard S, Guérard F, Chalopin B, Carlier T, Faivre-Chauvet A, et al. What is the Best Radionuclide for Immuno-PET of Multiple Myeloma? A Comparison Study Between (89)Zr- and (64)Cu-Labeled Anti-CD138 in a Preclinical Syngeneic Model. Int J Mol Sci. 2019;20(10). doi: 10.3390/ijms20102564 PMCID: PMC6567828 31137758 PMC6567828

[79] Sevillano AM, Aguilar-Calvo P, Kurt TD, Lawrence JA, Soldau K, Nam TH, et al. Prion protein glycans reduce intracerebral fibril formation and spongiosis in prion disease. J Clin Invest. 2020. doi: 10.1172/JCI131564 31985492 PMC7269597

