# Supplementary figures and images for: Neuronal Ndst1 depletion accelerates prion protein clearance and slows neurodegeneration in prion infection
Source: PLoS Pathog. 2023 Sep 25;19(9):e1011487. doi: 10.1371/journal.ppat.1011487 (PMC10586673; doi:10.1371/journal.ppat.1011487)

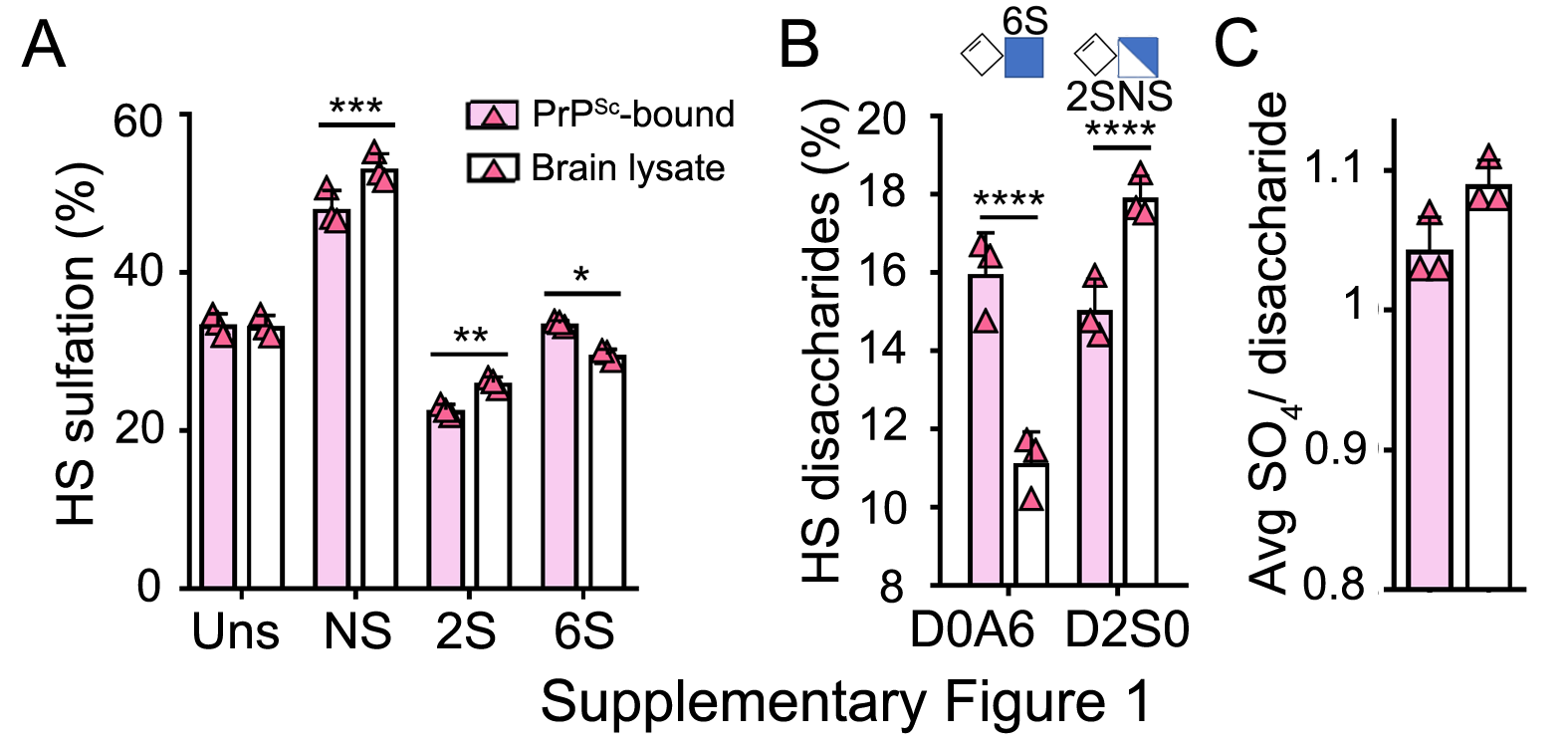

Supplement: S1 Fig — Mass spectrometry analysis of HS bound to PrPSc and brain lysate from the same sample (the former published [46]). Quantification shows (A), unsulfated (NAc and NH2) and sulfated (NS, 2S, 6S) HS, (B), individual HS disaccharides, and (C), the average sulfation per disaccharide of HS. N = 3 cerebellar samples. Note: other disaccharides were not significantly different (S4 Table). *P< 0.05, **P< 0.01, ***P< 0.005 and ****P< 0.001, one-way ANOVA with Tukey’s post test (panels A and B), and unpaired two-tailed t-test with Bonferroni’s post test (panel C). NAc: N-acetylglucosamine (GlcNAc); NH2: glucosamine (GlcNH2); NS: N-sulfated glucosamine (GlcNS); 2S: 2-O-sulfated glucuronic or iduronic acids (2-O-S); 6S: 6-O-sulfated glucosamine (6-O-S). (TIF) [file ppat.1011487.s001.tif]

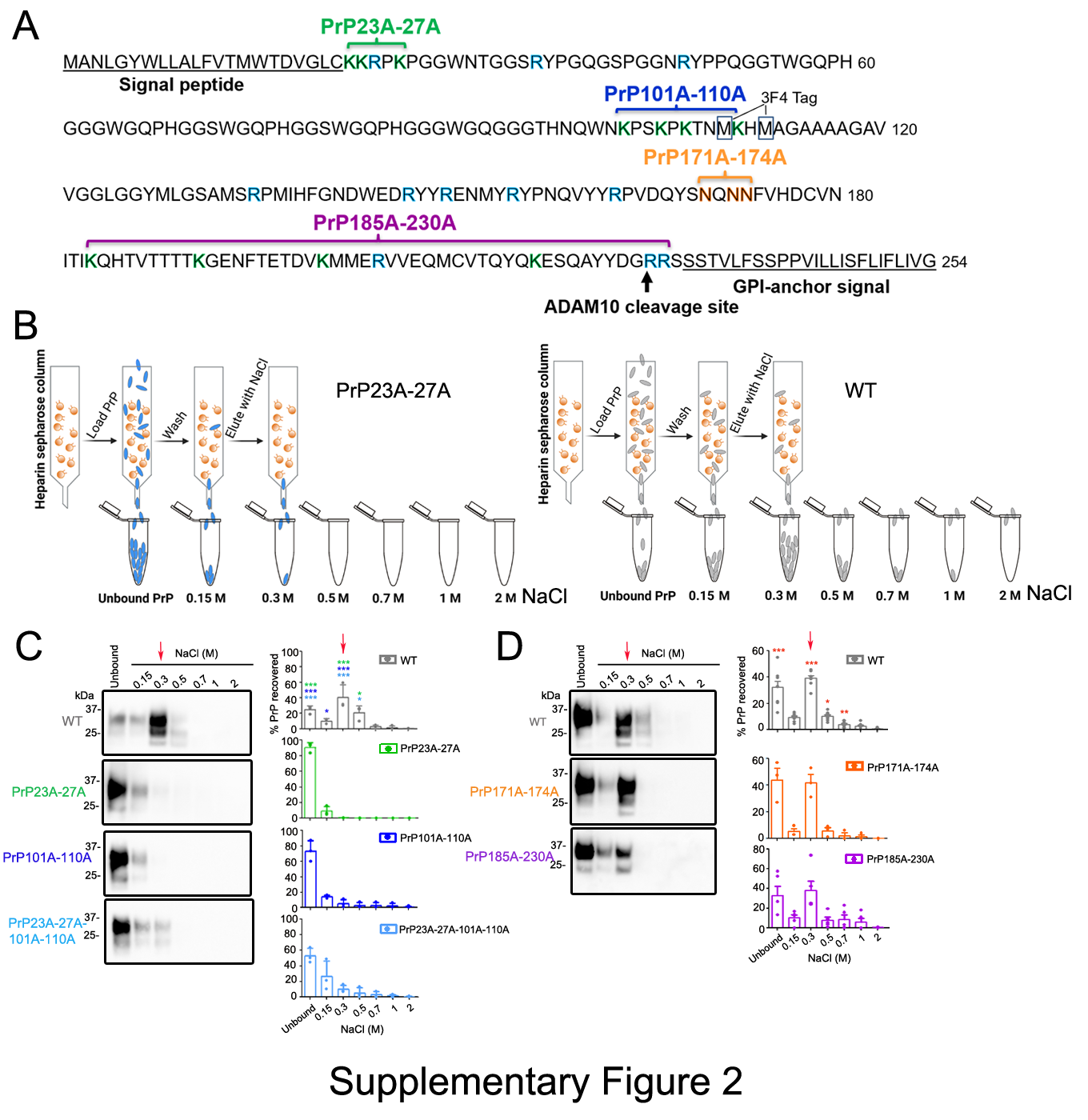

Supplement: S2 Fig — (A) Mouse PrP amino acid sequence with alanine substitutions highlighted (Lys-, Arg-, and Asn- to Ala). The 3F4 epitope tag detected by anti-PrP antibody 3F4 (Leu- to Met and Val- to Met) is also shown. (B) Schematic representation of the heparin sepharose chromatography columns packed with heparin-coated beads. Heparin-bound mutant and WT PrP were step eluted using increasing concentrations NaCl. (C, D) Representative immunoblots and quantification of PrP recovered after each NaCl elution to assess the heparin binding affinity of mouse WT PrP compared to mutant PrP. While 0.3 M of NaCl is sufficient to elute most of the WT PrP bound to heparin (red arrow), Lys- and Arg- to Ala PrP mutants either run through the column without binding heparin (unbound) or are eluted with 0.15 M NaCl (C). N = 3–4 samples per PrP mutant. *P< 0.05, **P< 0.01 and ***P< 0.005, Two-way ANOVA with Bonferroni’s post test (panels C and D). Panel B created with BioRender.com. (TIF) [file ppat.1011487.s002.tif]

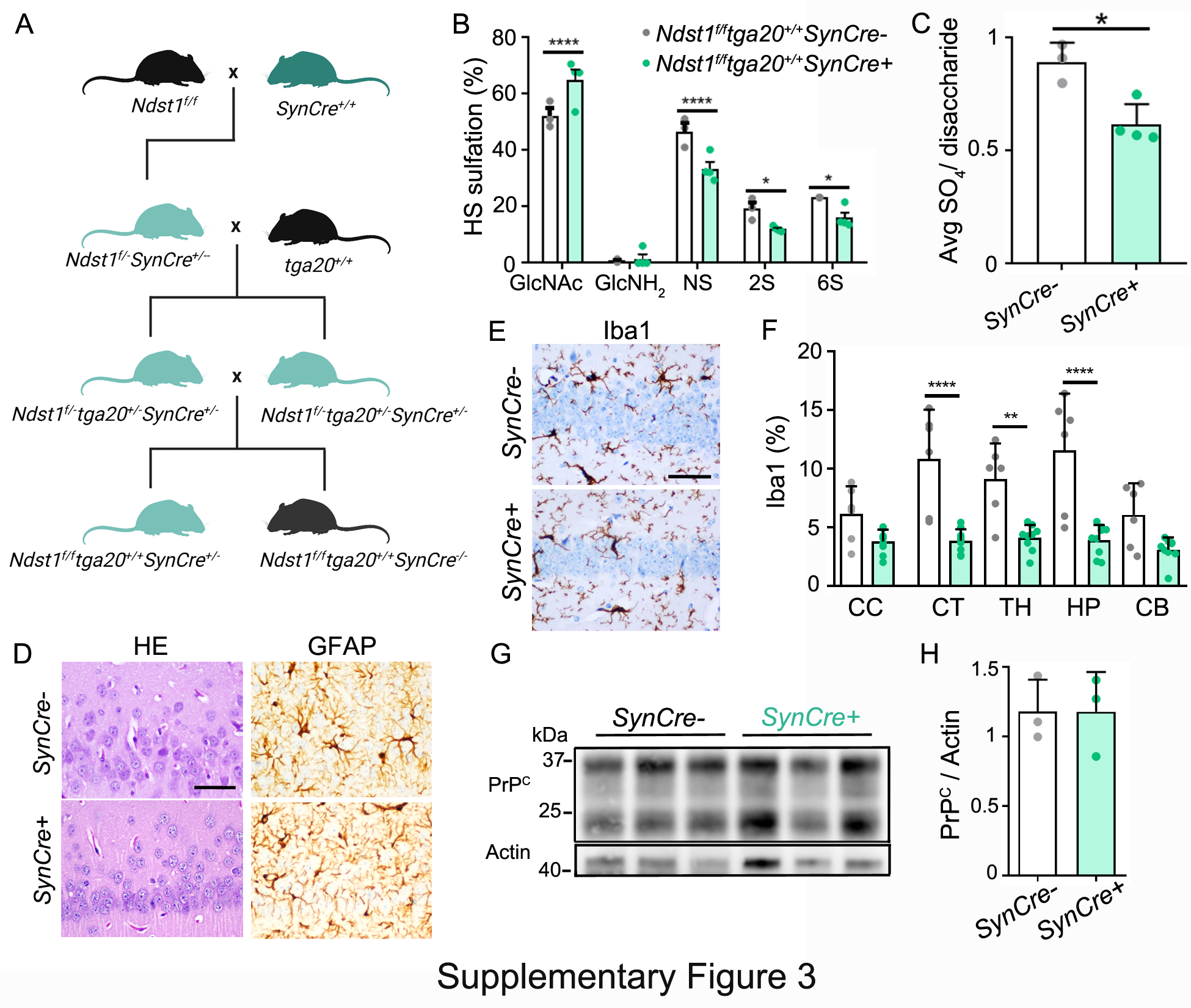

Supplement: S3 Fig — (A) Schematic representation of the breeding strategy to generate Ndst1f/ftga20+/+SynCre+/- mice and littermate controls. (B) HS in whole brain lysates from uninfected SynCre- and SynCre+ mice. Quantification of unsulfated (NAc and NH2) and sulfated (NS, 2S, 6S) HS and (C) average sulfation per HS disaccharide (n = 5 SynCre- and 6 SynCre+ brains). (D) Representative images of hematoxylin-eosin (HE)-stained, astrocyte (GFAP)-immunolabelled, and (E) microglia (Iba1)-immunolabelled brain sections (hippocampus). Scale bar = 100 μm. (F) Quantification of Iba1-immunolabelled over total area. N = 6 samples per group. CC: corpus callosum, CT: cortex, TH: thalamus, HP: hippocampus and CB: cerebellum. (G) PrP immunoblots and (H) quantification of PrPC levels in SynCre- and SynCre+ mice. N = 3 samples per group. *P< 0.05, **P< 0.01 and ****P< 0.001, two-way ANOVA with Bonferroni’s post test (panels B and F), and unpaired two-tailed t-test with Bonferroni’s post test (panels C and H). NAc: N-acetylglucosamine (GlcNAc); NH2: glucosamine (GlcNH2); NS: N-sulfated glucosamine (GlcNS); 2S: 2-O-sulfated glucuronic or iduronic acids (2-O-S); 6S: 6-O-sulfated glucosamine (6-O-S). Panel A created with BioRender.com. (TIF) [file ppat.1011487.s003.tif]

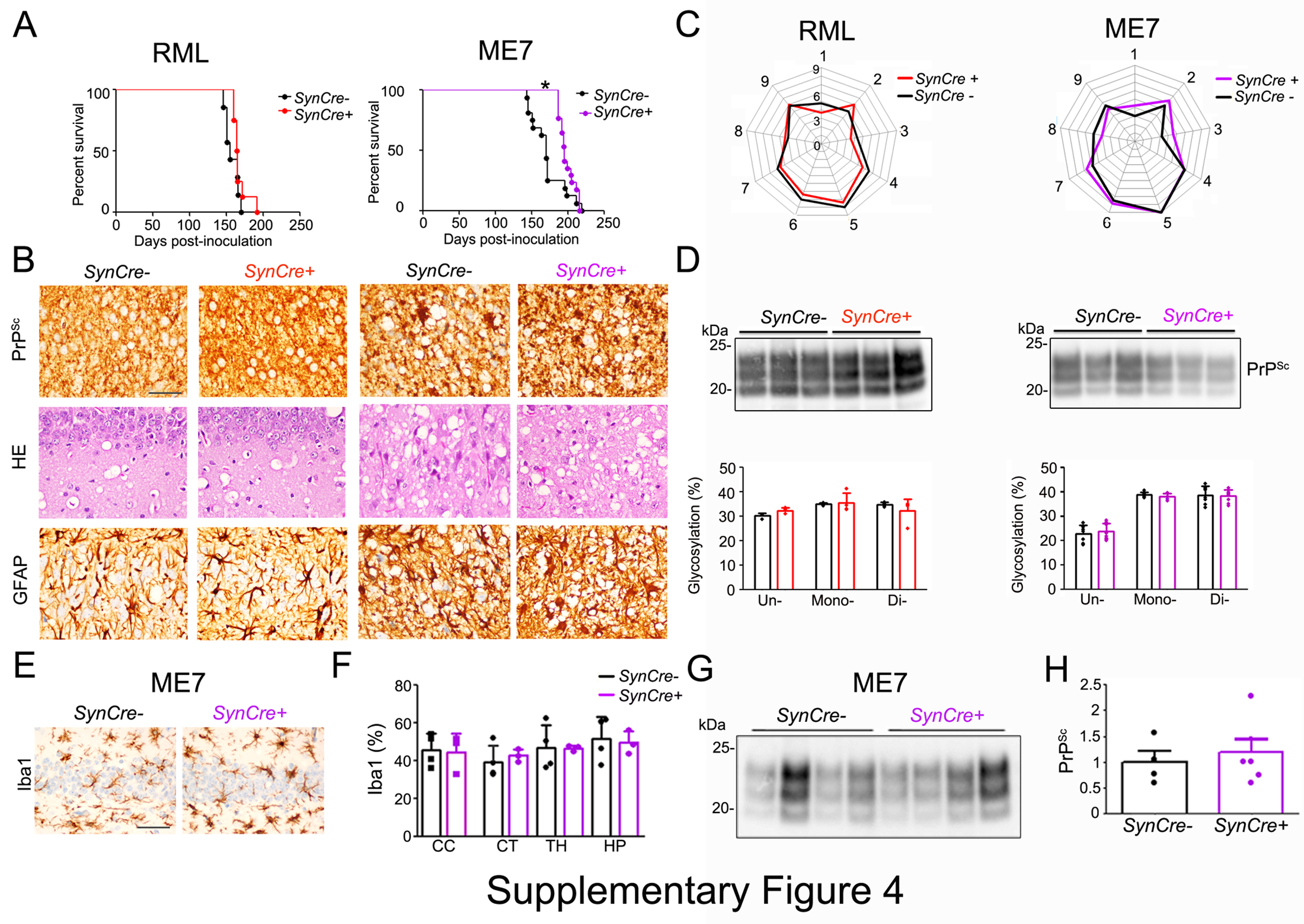

Supplement: S4 Fig — (A) Survival curves of SynCre- and SynCre+ mice intracerebrally inoculated with RML or ME7 prions (RML: n = 7 SynCre- and 8 SynCre+ mice; ME7: n = 16 SynCre- and 17 SynCre+ mice). (B) Brain sections (hippocampus) immunolabelled for PrPSc or GFAP or stained with hematoxylin and eosin (HE) show prion aggregate distribution and morphology, spongiform degeneration, and astrogliosis in SynCre- and SynCre+ brains. Scale bar = 50 μm. (C) Lesion profiles of RML- and ME7-infected SynCre- and SynCre+ mice (1-dorsal medulla, 2-cerebellum, 3-hypothalamus, 4-medial thalamus, 5-hippocampus, 6-septum, 7-cerebral cortex, 8- cerebral peduncle and 9- cerebellar peduncle) (RML: n = 5 mice per genotype; ME7: n = 6 SynCre- and 7 SynCre+ mice). (D) PrPSc immunoblots (post-PK digest) show electrophoretic mobility and glycoprofile of PK-digested PrPSc. N = 6 per genotype. (E) Immunolabelling for microglia (Iba1) (representative images of hippocampus) and (F) quantification of the Iba1-stained area in CC, CT, HP, TH. Scale bar = 50 μm. (G) Representative immunoblots for PrPSc (post PK digest) from 30 μg brain, and (H) quantification of PrPSc levels in the whole brain lysate at terminal disease. *P< 0.05, log-rank (Mantel-Cox) test (panel A), unpaired two-tailed t-test with Bonferroni’s post test (panels D and H), and two-way ANOVA with Bonferroni’s post test (panel F). CC: corpus callosum, CT: cerebral cortex, TH: thalamus and HP: hippocampus; PK: proteinase K. (TIF) [file ppat.1011487.s004.tif]

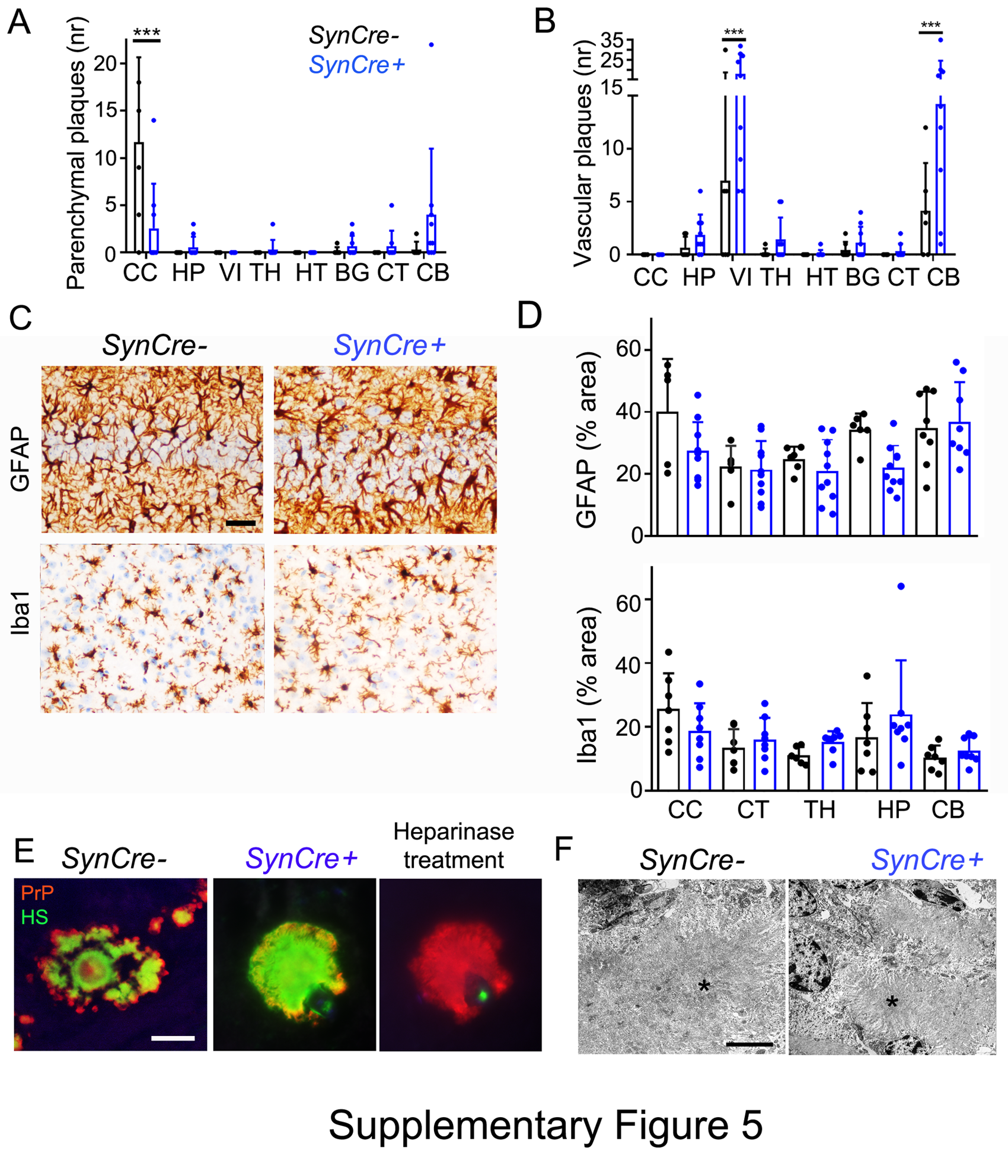

Supplement: S5 Fig — Quantification of (A) parenchymal and (B) vascular plaques in brain sections immunolabelled for PrP and endothelial cells (CD31) (representative sections shown in Fig 2G). N = 6 SynCre- and 9 SynCre+ mice. (C) Representative images of hippocampus from SynCre- and SynCre+ mice show immunolabeling for astrocytes (GFAP) and microglia (Iba1). (D) Quantification of the GFAP- and Iba1-immunolabelled area in C. Scale bar = 50 μm. N = 7 SynCre- mice and 8 SynCre+ mice. (E) Dual immunostaining of mCWD-infected brain sections for PrP and HS shows mCWD plaques label strongly for HS [corpus callosum (SynCre-) and thalamus (SynCre+)]. Pre-treating brain sections with heparinases abolished HS labelling. Scale bar = 50 μm. (F) Electron microscopy images of mCWD plaques in corpus callosum (SynCre-) and velum interpositum (SynCre+). The dense core is highlighted with an asterisk. Scale bar = 5 μm. ***P< 0.005, two-way ANOVA with Bonferroni’s post test (panels A, B and D). CC: corpus callosum, HP: hippocampus, VI: velum interpositum, TH: thalamus, HT: hypothalamus, BG: basal ganglia, CT: cerebral cortex, and CB: cerebellum. (TIF) [file ppat.1011487.s005.tif]

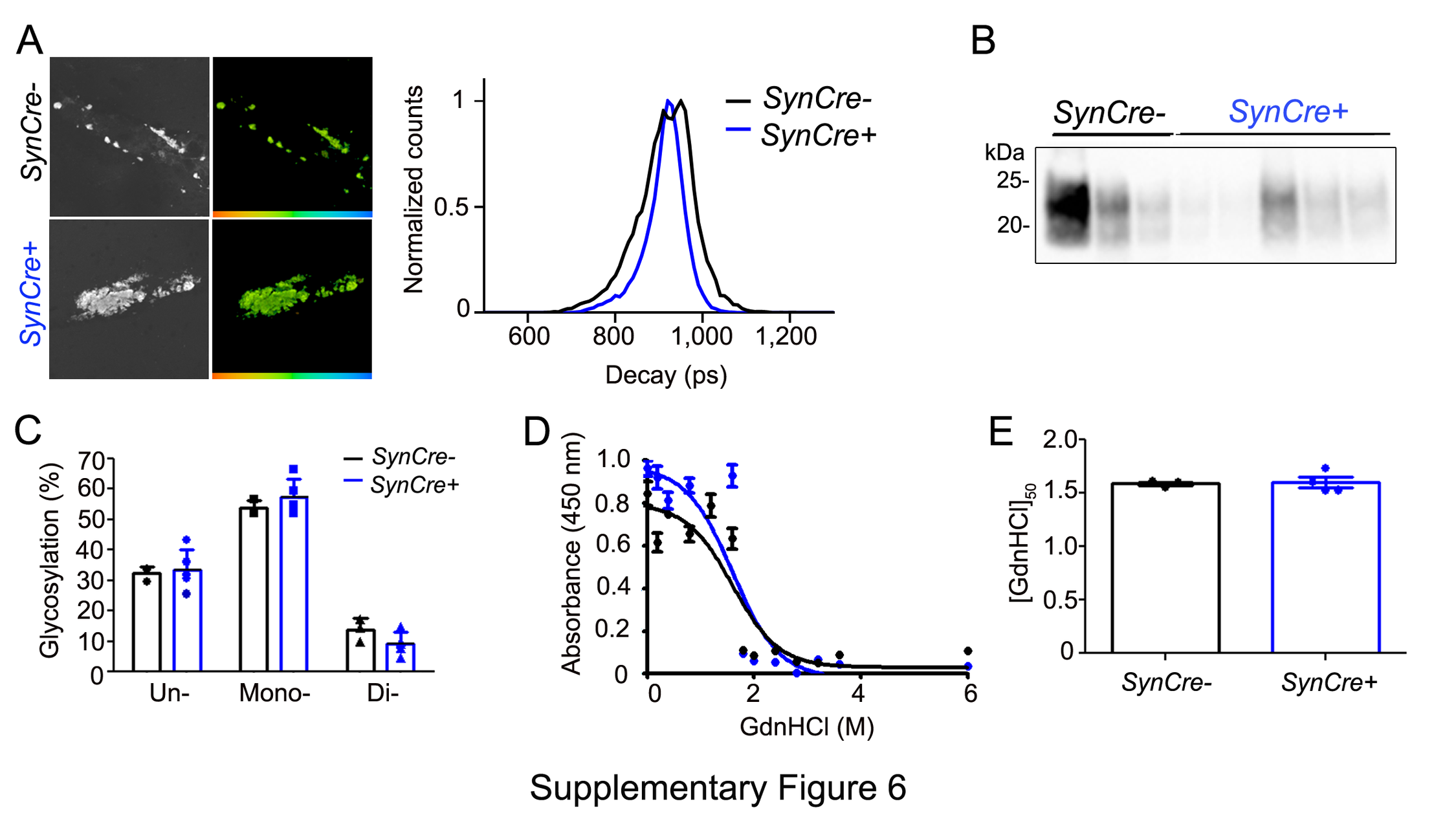

Supplement: S6 Fig — (A) Representative images and curves of the h-FTAA fluorescence life-time decay of mCWD prion plaques in Ndst1f/ftga20+/+SynCre- and SynCre+ brain sections. N = 5 SynCre- mice and 3 SynCre+ mice. (B) PrPSc (mCWD) electrophoretic mobility and (C) glycoprofile in SynCre- and SynCre+ mice. N = 6 SynCre- and 7 SynCre+ mice. (D) Representative example of PrPSc stability as measured by GdnHCl denaturation in mCWD-infected SynCre- and SynCre+ mice. N = 4 brain samples per genotype. (E) [GdnHCl]1/2 for PrPSc in SynCre- and SynCre+ brain. ps = picoseconds (panel A). Unpaired two-tailed t-test with Bonferroni’s post test (panels A, C, and E). (TIF) [file ppat.1011487.s006.tif]

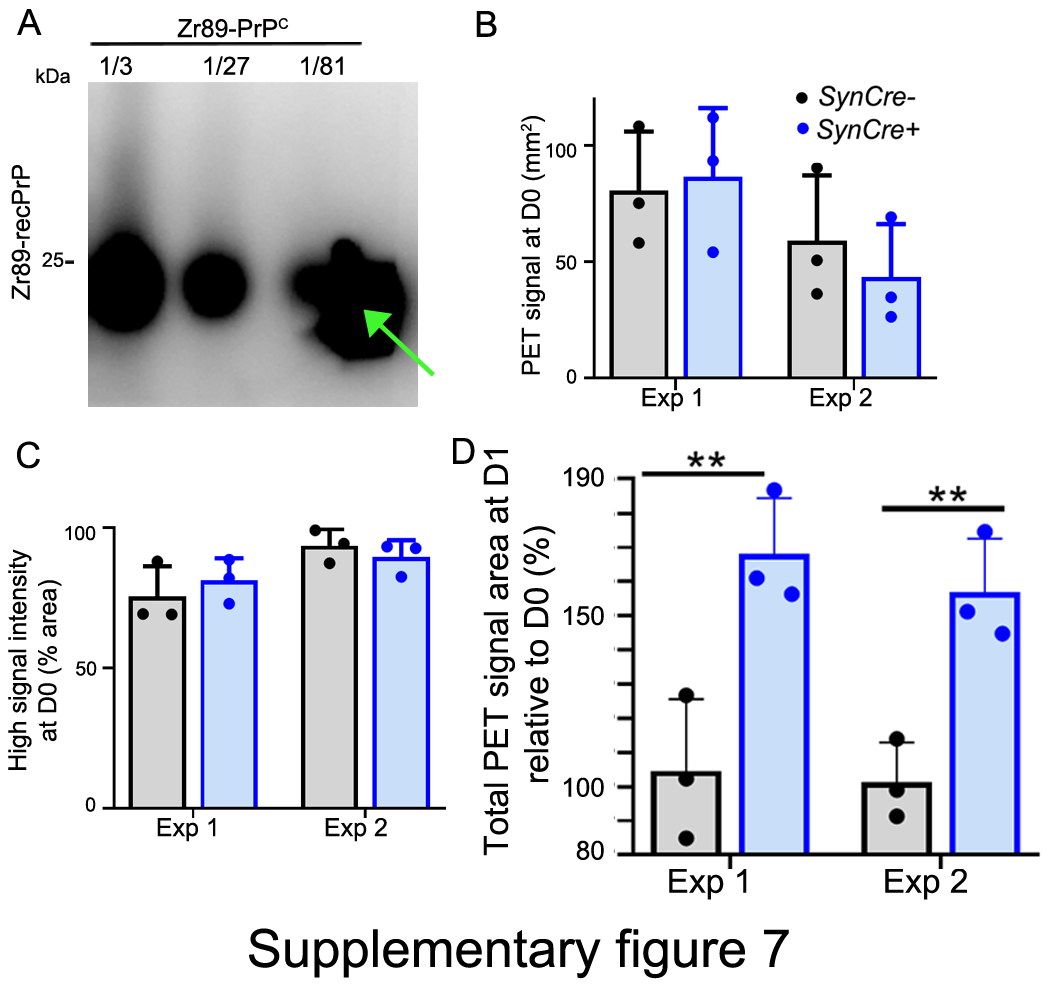

Supplement: S7 Fig — (A) Phosphor-imaged gel following electrophoresis of Zr89-recPrP (1:3 dilution series) four days after radiolabeling shows that recPrP was not degraded and remained radiolabeled. Zr89 was placed on the precision ladder at the expected size for recPrP (green arrow, 25 kDa). (B, C) Quantified signal from sagittal brain sections immediately after Zr89-recPrP injection (D0) show that (B) the total PET signal area and (C) the high signal intensity (>100 μCi) area for two experiments (Exp) are similar in SynCre- and SynCre+ mice. (D) Total PET signal area at day 1 (D1; 20 hours later) is 60 to 80% increased in SynCre+ brain versus only increased by 5% in SynCre- brain in two experiments. *P< 0.05 and **P< 0.01, two-way ANOVA with Bonferroni’s post test (panels B-D). N = 3 mice per genotype for each experiment. (TIF) [file ppat.1011487.s007.tif]

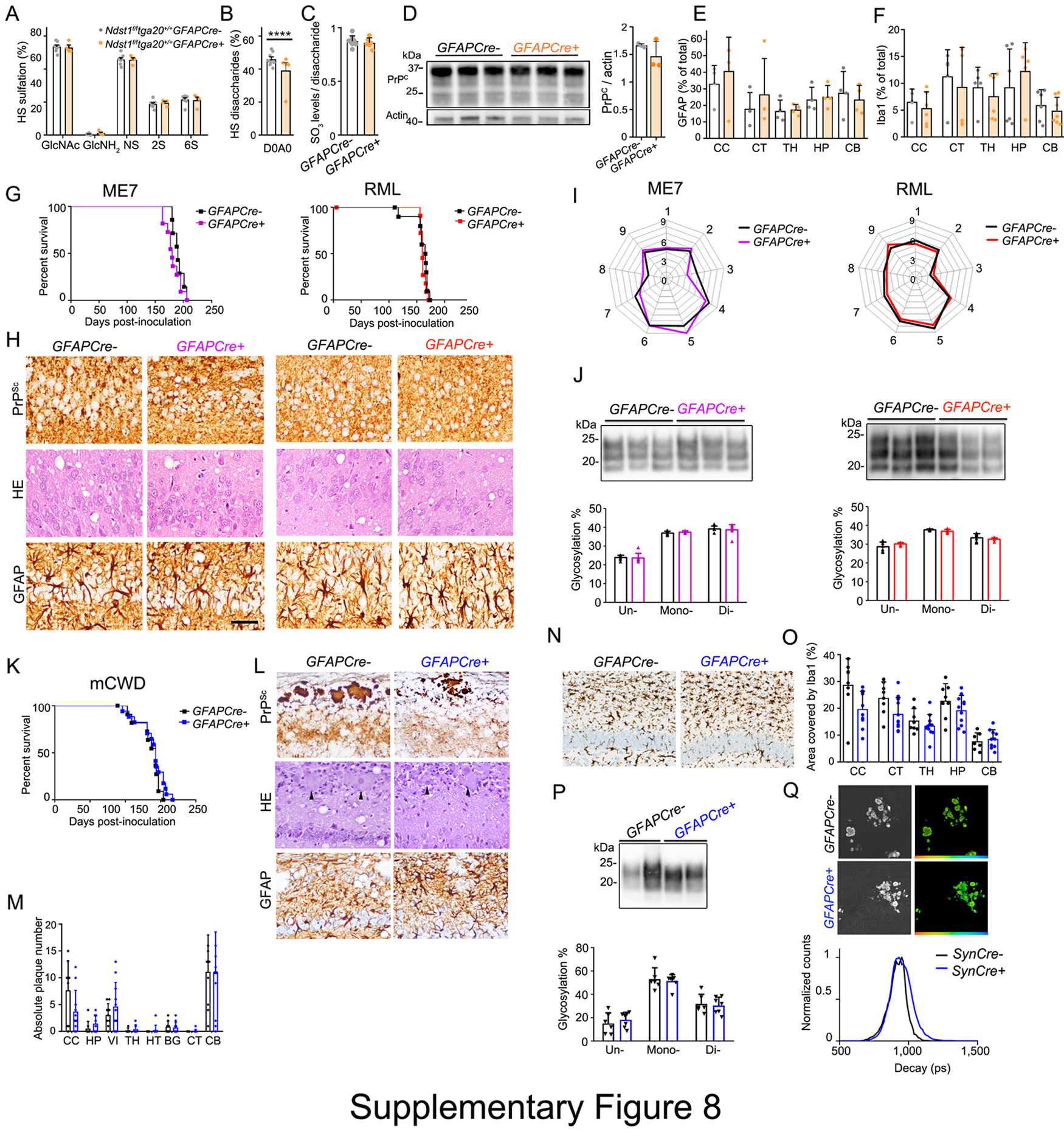

Supplement: S8 Fig — Quantification of (A) unsulfated (NAc and NH2) and sulfated (NS, 2S, 6S) HS, (B) individual HS disaccharides, and (C) average sulfation per HS disaccharide in whole brain lysates from approximately 420 day old Ndst1f/ftga20+/+GFAPCre- and Cre+ brain samples (n = 5 GFAPCre- and 6 Cre+ brain samples). Other disaccharides shown in S8 Table. (D) Immunoblotting for PrPC levels in GFAPCre+ and Cre- brain. N = 3 brains per genotype. (E) Quantification of GFAP- and (F) Iba1-immunolabelled area (n = 4–7 mice per group per stain). (G) Survival curves for ME7- and RML-inoculated Ndst1f/fGFAPCre+ and Cre- mice in ME7-inoculated Ndst1f/fGFAPCre+ (ME7: n = 7 GFAPCre- mice and 11 Cre+ mice; RML: n = 11 GFAPCre- and 10 Cre+ mice). (H) Brain sections (hippocampus) immunolabelled for PrP or GFAP, or stained with hematoxylin and eosin (HE), and (I) lesion profiles for ME7- and RML- infected GFAPCre+ brain (ME7: n = 5 GFAPCre- and 4 Cre+ mice; RML: n = 5 GFAPCre- and 6 Cre+ mice). (J) Electrophoretic mobility and glycoprofile of PrPSc from ME7- and RML-infected Ndst1f/GFAPCre- brain (ME7: n = 6 GFAPCre- and 8 Cre+ brains; RML: n = 8 brains per genotype). (K) mCWD-infected Ndst1f/ftga20+/+GFAPCre+ and Cre- mice survival times (n = 12 GFAPCre- and 16 Cre+ mice). (L) Spongiform change [HE stains (arrowheads depict plaques), PrPSc, and astrocyte immunolabelling (GFAP)], (M) plaque distribution (n = 9 GFAPCre- and 11 Cre+ brains), and (N) microglial response (n = 8 GFAPCre- and 11 GFAPCre+ brains), the latter quantified in (O) in GFAPCre+ and Cre- mice. (P) The electrophoretic mobility, glycoprofile (n = 6 GFAPCre- and 7 Cre+ brains) and (Q) PrPSc-bound h-FTAA fluorescence life-time decay in brain (n = 5 GFAPCre- and 6 Cre+). Two-way ANOVA with Bonferroni’s post test (panels A-C, E, F, M and O), and unpaired two-tailed t-test with Bonferroni’s post test (panels D, J, P and Q), and Log-rank (Mantel-Cox) test (panel G and K). NAc: N-acetylglucosamine (GlcNAc); NH2: glucosamine (GlcNH2); NS: N [file ppat.1011487.s008.tif]
