# Supplementary material for: Neuronal Ndst1 depletion accelerates prion protein clearance and slows neurodegeneration in prion infection
Source: PLoS Pathog. 2023 Sep 25;19(9):e1011487. doi: 10.1371/journal.ppat.1011487 (PMC10586673; doi:10.1371/journal.ppat.1011487)
Supplement: S1 Table — (PDF) [file ppat.1011487.s009.pdf]

S1 Table. Disaccharide composition of heparan sulfate molecules bound to PrP^{Sc} versus brain lysate of ME7-infected mice

**Prion strain**	ME7							
**Disaccharide (%)**	HS bound to PrP^{Sc}			**Mean ± SEM**	HS in brain			**Mean ± SEM**
**D0H0**	0	0	0	**0 ± 0**	2.4	2.3	0	**1.6 ± 1**
**D0A0**	36	41	36	**38 ± 2**	49	51	49	**49 ± 1**
**D0H6**	0	0.03	0	**0.01 ± 0**	0.24	0.17	0.02	**0.14 ± 0**
**D2H0**	0	0	0	**0 ± 0**	0.13	0.08	0	**0.07 ± 0**
**D0S0**	18	19	19	**19 ± 0**	16	16	19	**17 ± 1**
**D0A6**	9.1	8.4	9.5	**9 ± 0**	7.3	7.2	5.2	**6.6 ± 1**
**D2A0**	0.97	1.1	0.94	**1 ± 0**	0.96	0.93	1.4	**1.1 ± 0**
**D2H6**	0	0	0	**0 ± 0**	0.01	0.01	0	**0.01 ± 0**
**D0S6**	9.5	8.4	12	**10 ± 1**	6.1	6.28	4.35	**5.6 ± 1**
**D2S0**	19	17	16	**17 ± 1**	13	12	17	**14 ± 2**
**D2A6**	0	0	0.01	**0 ± 0**	0.04	0.03	0	**0.02 ± 0**
**D2S6**	7.8	5.7	6.9	**6.8 ± 1**	4.8	4.6	4.2	**4.5 ± 0**
