# Supplementary material for: Neuronal Ndst1 depletion accelerates prion protein clearance and slows neurodegeneration in prion infection
Source: PLoS Pathog. 2023 Sep 25;19(9):e1011487. doi: 10.1371/journal.ppat.1011487 (PMC10586673; doi:10.1371/journal.ppat.1011487)
Supplement: S2 Table — (PDF) [file ppat.1011487.s010.pdf]

S2 Table. Disaccharide composition of heparan sulfate molecules bound to PrP^{Sc} versus brain lysate of mCWD-infected mice

**Prion strain**	**mCWD**							
**Disaccharide (%)**	HS bound to PrP^{Sc}				**Mean ± SEM**	HS in brain			**Mean ± SEM**
**D0H0**	0	0	0	0	**0 ± 0**	0.07	0.37	0	**0.15 ± 0**
**D0A0**	42	46	43	39	**42 ± 2**	62	47	47	**52 ± 5**
**D0H6**	0.13	0	0	0.15	**0.07 ± 0**	0	0	0	**0 ± 0**
**D2H0**	0.06	0	0	0.02	**0.02 ± 0**	0	0	0	**0 ± 0**
**D0S0**	21	21	20	19	**20 ± 0**	13	15	19	**16 ± 2**
**D0A6**	11	11	11	12	**11 ± 0**	13	7.7	7.2	**9.2 ± 2**
**D2A0**	0.1	0.21	0.72	0.64	**0.42 ± 0**	0.02	0.93	0.76	**0.57 ± 0**
**D2H6**	0	0	0	0	**0 ± 0**	0	5.	0	**2 ± 2**
**D0S6**	7.5	6.3	7	8.9	**7.4 ± 1**	8.5	11	11	**10 ± 1**
**D2S0**	16	11	13	15	**14 ± 1**	3.1	11	9.2	**7.6 ± 2**
**D2A6**	0.02	0	0.1	0	**0.03 ± 0**	0.05	1.2	5.9	**2.4 ± 2**
**D2S6**	4	5	5.7	6	**5.1 ± 0**	0.04	0.47	0.02	**0.18 ± 0**
