# Supplementary material for: Neuronal Ndst1 depletion accelerates prion protein clearance and slows neurodegeneration in prion infection
Source: PLoS Pathog. 2023 Sep 25;19(9):e1011487. doi: 10.1371/journal.ppat.1011487 (PMC10586673; doi:10.1371/journal.ppat.1011487)
Supplement: S3 Table — (PDF) [file ppat.1011487.s011.pdf]

S3 Table. Disaccharide composition of heparan sulfate molecules bound to PrP^{Sc} versus brain lysate of sCJD patients

**Prion**	**sCJD**							
**Disaccharide (%)**	HS bound to PrP^{Sc}			**Mean ± SEM**	HS in brain			**Mean ± SEM**
**D0H0**	0	0	0	**0 ± 0**	2.4	2.6	1.7	**2.2 ± 0**
**D0A0**	32	40	37	**37 ± 2**	45	43	45	**44 ± 1**
**D0H6**	0	0.08	0	**0.03 ± 0**	0.39	0.48	0.49	**0.46 ± 0**
**D2H0**	0	0	0	**0 ± 0**	0.02	0	0	**0.01 ± 0**
**D0S0**	14	16	14	**15 ± 1**	15	15	14	**15 ± 0**
**D0A6**	18	16	16	**16 ± 1**	8.4	9.3	9.05	**8.9 ± 0**
**D2A0**	0.56	0.19	0.07	**0.27 ± 0**	0.82	0.59	0.68	**0.7 ± 0**
**D2H6**	0.28	0.17	0.44	**0.3 ± 0**	0.06	0.07	0.07	**0.07 ± 0**
**D0S6**	10	10	9.3	**10 ± 0**	7	8.9	9	**8 ± 1**
**D2S0**	9.6	9	8.4	**9 ± 0**	11	10	9.4	**10 ± 1**
**D2A6**	0.04	0.15	0	**0.06 ± 0**	0	0	0	**0 ± 0**
**D2S6**	15	8.3	16	**13 ± 2**	10	10	11	**10 ± 0**
