# Supplementary material for: Neuronal Ndst1 depletion accelerates prion protein clearance and slows neurodegeneration in prion infection
Source: PLoS Pathog. 2023 Sep 25;19(9):e1011487. doi: 10.1371/journal.ppat.1011487 (PMC10586673; doi:10.1371/journal.ppat.1011487)
Supplement: S4 Table — (PDF) [file ppat.1011487.s012.pdf]

**S4 Table.** Disaccharide composition of heparan sulfate molecules bound to $PrP^{Sc}$ versus brain lysate of GSS patients

| **Prion** | **GSS** | | | | | | | |
|---|---|---|---|---|---|---|---|---|
| **Disaccharide (%)** | HS bound to $PrP^{Sc}$ | | | **Mean ± SEM** | HS in brain | | | **Mean ± SEM** |
| **D0H0** | 4 | 3.1 | 2.9 | **3.2 ± 0** | 4 | 3.7 | 3.4 | **3.7 ± 0** |
| **D0A0** | 30 | 29 | 32 | **30 ± 1** | 29 | 28 | 31 | **30 ± 1** |
| **D0H6** | 0.92 | 0.79 | 0.73 | **0.81 ± 0** | 0.84 | 0.75 | 0.72 | **0.77 ± 0** |
| **D2H0** | 0.21 | 0.21 | 0.22 | **0.21 ± 0** | 0.16 | 0.19 | 0.18 | **0.18 ± 0** |
| **D0S0** | 17 | 18 | 16 | **17 ± 1** | 18 | 19 | 16 | **18 ± 1** |
| **D0A6** | 17 | 15 | 16 | **16 ± 1** | 12 | 10 | 12 | **11 ± 1** |
| **D2A0** | 1.1 | 1.1 | 0.92 | **1 ± 0** | 1.3 | 1.3 | 1 | **1.2 ± 0** |
| **D2H6** | 0.13 | 0.09 | 0.22 | **0.15 ± 0** | 0.05 | 0.05 | 0.12 | **0.07 ± 0** |
| **D0S6** | 10 | 12 | 9.9 | **10 ± 1** | 11 | 11 | 10 | **11 ± 0** |
| **D2S0** | 14 | 16 | 15 | **15 ± 1** | 18 | 19 | 18 | **18 ± 0** |
| **D2A6** | 0.28 | 0.22 | 0.19 | **0.23 ± 0** | 0.23 | 0.19 | 0.13 | **0.18 ± 0** |
| **D2S6** | 5.8 | 5.8 | 6.3 | **6 ± 0** | 6 | 7 | 7.3 | **6.6 ±0** |
