# Supplementary material for: Neuronal Ndst1 depletion accelerates prion protein clearance and slows neurodegeneration in prion infection
Source: PLoS Pathog. 2023 Sep 25;19(9):e1011487. doi: 10.1371/journal.ppat.1011487 (PMC10586673; doi:10.1371/journal.ppat.1011487)
Supplement: S5 Table — (PDF) [file ppat.1011487.s013.pdf]

S5 Table. Disaccharide composition of heparan sulfate molecules in uninfected *Ndst1$^{f/f}$tga20$^{+/+}$SynCre*- and *SynCre*+ brain

Disaccharide (%)	*Ndst1$^{f/f}$tga20$^{+/+}$SynCre-*				Mean ± SEM	*Ndst1$^{f/f}$tga20$^{+/+}$SynCre+*				Mean ± SEM
**D0H0**	1.1	0.77	0.22	0	**0.51 ± 0**	0	0	5.2	1.5	**1.7 ± 1**
**D0A0**	48	43	41	51	**46 ± 2**	61	60	46	36	**51 ± 6**
**D0H6**	0.33	0.07	0.04	0	**0.11 ± 0**	0	0	0.7	0.7	**0.35 ± 0**
**D2H0**	0.01	0.01	0.01	0	**0.01 ± 0**	0	0	0.01	0	**0 ± 0**
**D0S0**	18	20	20	19	**19 ± 0**	18	17	20	27	**20 ± 2**
**D0A6**	8.5	7.5	6.7	6	**7.2 ± 1**	7.1	7.3	7.5	4.5	**6.6 ± 1**
**D2A0**	0.82	0.99	1.3	0.06	**0.8 ± 0**	0.03	0	0.61	1.04	**0.42 ± 0**
**D2H6**	0.02	0	0.07	0.02	**0.03 ± 0**	0.02	0.02	0.19	0	**0.1 ± 0**
**D0S6**	8.4	9.5	10	4.7	**8.1 ± 1**	3.1	3.9	8.3	11	**6.6 ± 2**
**D2S0**	8.5	12	15	15	**13 ± 2**	7.8	8.2	7.5	10	**8.4 ± 1**
**D2A6**	0	0	0	0	**0**	0.01	0	0.02	0	**0.01 ± 0**
**D2S6**	5.9	6.3	6.6	4.6	**5.8 ± 0**	3.4	3.4	5	8.03	**5 ± 1**
