# Supplementary material for: Neuronal Ndst1 depletion accelerates prion protein clearance and slows neurodegeneration in prion infection
Source: PLoS Pathog. 2023 Sep 25;19(9):e1011487. doi: 10.1371/journal.ppat.1011487 (PMC10586673; doi:10.1371/journal.ppat.1011487)
Supplement: S6 Table — (PDF) [file ppat.1011487.s014.pdf]

S6 Table. Quantification of mCWD parenchymal and vascular plaques in mCWD-infected *Ndst1*$^{f/f}$*tga20*$^{+/+}$*SynCre*- and *SynCre*+ brain

	**Mouse genotype**	
**Brain area**	***Ndst1*$^{f/f}$*SynCre-***	***Ndst1*$^{f/f}$*SynCre+***
**Corpus callosum**	12 ± 4*	3 ± 2
**Velum interpositum**	3 ± 1	17 ± 3
**Medial thalamus**	0	1 ± 1
**Hippocampus**	1 ± 0	2 ± 1
**Basal ganglia**	0	1 ± 1
**Cerebral peduncles**	0	0
**Cerebellum**	4 ± 4	14 ± 3
**Cerebellar peduncles**	0	0
**Medulla**	0	0

* Standard error of the mean
