# Supplementary material for: Neuronal Ndst1 depletion accelerates prion protein clearance and slows neurodegeneration in prion infection
Source: PLoS Pathog. 2023 Sep 25;19(9):e1011487. doi: 10.1371/journal.ppat.1011487 (PMC10586673; doi:10.1371/journal.ppat.1011487)
Supplement: S7 Table — (PDF) [file ppat.1011487.s015.pdf]

S7 Table. mCWD seeding activity in the spinal cord of *Ndst1*$^{f/f}$*tga20*$^{+/+}$*SynCre*- and *SynCre*+ mice using real-time quaking-induced conversion

	Spinal cord samples	
**RT-QuIC outcomes**	***Ndst1***$^{f/f}$***SynCre-***	***Ndst1***$^{f/f}$***SynCre+***
**Positive**	7	14
**Negative**	4	2
