# Supplementary material for: Neuronal Ndst1 depletion accelerates prion protein clearance and slows neurodegeneration in prion infection
Source: PLoS Pathog. 2023 Sep 25;19(9):e1011487. doi: 10.1371/journal.ppat.1011487 (PMC10586673; doi:10.1371/journal.ppat.1011487)
Supplement: S8 Table — (PDF) [file ppat.1011487.s016.pdf]

S8 Table. Disaccharide composition of heparan sulfate molecules in *Ndst1*$^{f/f}$*tga20*$^{+/+}$*GFAPCre-* and *GFAPCre+* brain

**Disaccharide (%)**	*Ndst1*$^{f/f}$*tga20*$^{+/+}$*GFAPCre-*				**Mean ± SEM**	*Ndst1*$^{f/f}$*tga20*$^{+/+}$*GFAPCre+*				**Mean ± SEM**
**D0H0**	0	0.3	0.45	0.44	**0.3 ± 0**	0.39	3.7	2.6	0.13	**1.8 ± 1**
**D0A0**	52	45	44	44	**46 ± 2**	43	20	43	46	**38 ± 6**
**D0H6**	0.03	0.19	0.22	0.3	**0.2 ± 0**	0.2	0.58	0.33	0.04	**0.31 ± 0**
**D2H0**	0	0	0	0.01	**0 ± 0**	0.01	0	0	0	**0 ± 0**
**D0S0**	19	21	21	20	**20 ± 1**	19	33	21	21	**24 ± 3**
**D0A6**	5.9	8	8.1	8.01	**7.5 ± 1**	7.6	1.8	7.1	7.03	**6 ± 1**
**D2A0**	0.11	0.98	0.88	0.84	**0.7 ± 0**	1.02	1.3	0.7	0.87	**0.94 ± 0**
**D2H6**	0.01	0	0	0.03	**0.01 ± 0**	0.03	0.11	0.03	0.02	**0.04 ± 0**
**D0S6**	5.6	6.6	7.01	9.3	**7.1 ± 1**	9.3	13	9.2	8.8	**9.4 ± 1**
**D2S0**	12	12	12	11	**12 ± 0**	13	15	10	12	**12 ± 1**
**D2A6**	0	0.03	0.06	0	**0.02 ± 0**	0.03	0.02	0	0	**0 ± 0**
**D2S6**	5.2	6.1	7.02	6.07	**6.1 ± 0**	6.8	11	6.5	6.5	**7.2 ± 1**
